# Supplementary material for: Prevalence and factors associated with tuberculosis among the mining communities in Mererani, Tanzania
Source: PLoS One. 2023 Mar 15;18(3):e0280396. doi: 10.1371/journal.pone.0280396 (PMC10016659; doi:10.1371/journal.pone.0280396)
Supplement: S1 File — (ZIP) [file pone.0280396.s001.zip › Supporting Information/S4_Abstract.docx]

# Abstract

Tuberculosis (TB) is among diseases of global health importance with Sub Saharan Africa (SSA) accounting for 25% of the global TB burden. TB prevalence among miners in SSA is estimated at 3,000-7,000/100,000, which is about 3 to 10-times higher than in the general population. The study’s objective was to determine the prevalence of TB and associated risk factors among mining communities in Mererani, northern Tanzania.

This was a cross-sectional study conducted from April 2019 to November 2021 involving current Small Scale Miners (SSM) and the General Community (GC). A total of 660 participants, 330 SSM and 330 GC were evaluated for the presence of TB. Data were analysed using Statistical Package for the Social Sciences (SPSS) database (IBM SPSS Statistics Version 27.0.0.0). Binary logistic regression (Generalized Linear Mixed Model) was used to determine the association between TB and independent predictors.

Prevalence of TB was 7%, about 27-times higher than the national prevalence of 0.25%. Participants from the general community had higher prevalence of TB 7.9% than SSM (6.1%). Both for SSM and the GC, TB was found to be associated with: lower education level (aOR=3.62, 95%CI=1.16-11.28), previous lung disease (aOR=4.30, 95%CI=1.48-12.53) and having symptoms of TB (aOR=3.24, 95%CI= 1.38-7.64). Specifically for the SSM, TB was found to be associated with Human Immunodeficiency Virus (HIV) infection (aOR=8.28, 95%CI=1.21-56.66).

Though significant progress has been attained in the control of the TB epidemic in Tanzania, still hot spots with significantly high burden of TB exists, including miners. More importantly, populations surrounding the mining areas, are equally affected, and needs more engagement in the control of TB so as to realize the Global End TB targets of 2035.

**Key Words**: tuberculosis, tanzanite mining, Tanzania
