# Supplementary material for: Prevalence and factors associated with tuberculosis among the mining communities in Mererani, Tanzania
Source: PLoS One. 2023 Mar 15;18(3):e0280396. doi: 10.1371/journal.pone.0280396 (PMC10016659; doi:10.1371/journal.pone.0280396)
Supplement: S1 File — (ZIP) [file pone.0280396.s001.zip › Supporting Information/S3_StudyProtocol.docx]

SILICA AND RADON EXPOSURES AND ITS ASSOCIATED EFFECTS ON RESPIRATORY SYSTEM AMONG SMALL SCALE TANZANITE MINERS IN MERERANI

By

Alexander William Mbuya

TUMA/KCMUCo/PHD/0051

A research proposal submitted in partial fulfillment of requirement for the requirements for Degree of Doctor of Philosophy (PhD) of Tumaini University Makumira

Kilimanjaro Christian Medical University College

Tumaini University Makumira

December 2018

Table of Contents

[DECLARATION iv](#_Toc92300909)

[CERTIFICATION v](#_Toc92300910)

[ACKNOWLEDGMENT v](#_Toc92300911)

[ABBREVIATIONS/ ACRONYMS vi](#_Toc92300912)

[DEFINITION OF TERMS viii](#_Toc92300913)

[ABSTRACT x](#_Toc92300914)

[CHAPTER ONE 1](#_Toc92300915)

[1. Introduction 1](#_Toc92300916)

[1.1. Background 1](#_Toc92300917)

[1.2. Literature Review 8](#_Toc92300918)

[1.3. Statement of the problem 25](#_Toc92300919)

[1.4. Study Justification 27](#_Toc92300920)

[1.5. Study hypotheses 27](#_Toc92300921)

[CHAPTER TWO 28](#_Toc92300922)

[2. Objectives 28](#_Toc92300923)

[2.1 Broad objective 28](#_Toc92300924)

[2.2 Specific objectives 28](#_Toc92300925)

[CHAPTER THREE 29](#_Toc92300926)

[3. Methodology 29](#_Toc92300927)

[3.1. Study design 29](#_Toc92300928)

[3.2. Study site 29](#_Toc92300929)

[3.3. Study population/area 30](#_Toc92300930)

[3.4. Eligibility criteria 31](#_Toc92300931)

[3.5. Sample Size & Sampling 31](#_Toc92300932)

[3.6. Study variables 32](#_Toc92300933)

[3.7. Data tools & Study procedures 33](#_Toc92300934)

[3.8. Data management & Analysis plan 36](#_Toc92300935)

[3.9. Ethical consideration 36](#_Toc92300936)

[3.10. Plan of result dissemination 37](#_Toc92300937)

[3.11. Study limitations 38](#_Toc92300938)

[PLAN OF ACTION 38](#_Toc92300939)

[BUDGET 40](#_Toc92300940)

[REFERENCES 42](#_Toc92300941)

[Annexes 48](#_Toc92300942)

[Data Collection Tools 48](#_Toc92300943)

[Interview Schedule – English Version 48](#_Toc92300944)

[Interview Schedule – Swahili Version 55](#_Toc92300945)

[In-depth Interview Guide – English Version 62](#_Toc92300946)

[In-depth Interview Guide – Swahili Version 65](#_Toc92300947)

[Consent Forms 68](#_Toc92300948)

[General Consent Form – English Version 68](#_Toc92300949)

[General Consent Form – Swahili Version 70](#_Toc92300950)

[In-depth Interview Consent Form – English Version 72](#_Toc92300951)

[In-depth Interview Consent Form – Swahili Version 74](#_Toc92300952)

# DECLARATION

I, **Alexander William Mbuya**, declare this research proposal to be my own original work and is being submitted for the partial fulfilment of the degree of Doctor of Philosophy of the Kilimanjaro Christian Medical University College (KCMUCo). It has never previously submitted for a degree or examination at the above mentioned university or any other university.

20^th^ December 2018

# CERTIFICATION

The undersigned certifies that this research proposal is the work of the student (**Alexander William Mbuya**) to be done as part of his training for a Doctor of Philosophy degree under my direct supervision.

The undersigned certifies that they have read and hereby recommends for consideration by KCMU College of the Tumaini University Makumira the proposal entitled:

‘SILICA AND RADON EXPOSURES AND ITS ASSOCIATED EFFECTS ON RESPIRATORY SYSTEM AMONG SMALL SCALE TANZANITE MINERS IN MERERANI’

in fulfilment of the requirement for the Degree of Doctor of Philosophy of the Tumaini University Makumira, Kilimanjaro Christian Medical University College.

1. Student’s signature

_____________________ ____________________

Alexander William Mbuya Date

1. Supervisor’s signature

Prof. Sia Msuya, PhD, Institute of Public Heatlh, KCMUCo

__________________________

Date

__________________________

Date

__________________________

Date

1. Co-supervisors


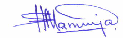


Dr. Simon Mamuya,

Muhimbili University College of Health and Allied Sciences

________________________

Dr. Hadija Semvua,

Kilimanjaro Clinical Research Institute

# ACKNOWLEDGMENT

I would like to acknowledge the continuous and tireless guidance and follow up from Prof. Sia Msuya, who is also my main supervisor, in realization of this important part of attaining my degree of the Doctor of Philosophy. My sincere appreciations to my co-supervisors: Dr. Simon Mamuya, of the Muhimbili University of Health and Allied Sciences (MUHAS) and Dr. Zumbi Musiba of the Barrick Gold Corporation, Tanzania for their continuous technical advices on key aspects of this research work, in particular on conduction of dust and radon gas sampling and measurements. I would also like to convey my work of thanks to my co-supervisor, Dr. Hadija Semvua of the Kilimanjaro Clinical Research Institute (KCRI) for her guidance and advice. Last though not least, my gratitude goes to Dr. David Barnes of the Wits Health Consortium (WHC) for his advice and sharing of key reference documents.

# ABBREVIATIONS/ ACRONYMS

ACGIH American Conference of Governmental Industrial Hygienists AIDS Acquired Immunodeficiency Syndrome

ASGM Artisanal Small-scale Gold Miners

ASSM Artisanal Small Scale Miners

ATS American Thoracic Society

BOHS British Occupational Hygiene Society

CO Carbon Monoxide

COPD Chronic Obstructive Pulmonary Diseases

DNA Dioxyribo-Nucleic Acid

ERS European Respiratory Society

GDP Gross Domestic Product

GOLD Global Initiative for Chronic Obstructive Lung Disease

HCP Health Care Providers

HIV Human Immunodeficiency Virus

ICRP International Agency for Research on Cancer

IDI In-Depth Interview

ILO International Labour Organization

IOM International Organization for Migration

JEM Job Exposure Matrix

KCMUCo Kilimanjaro Christian Medical University College

KCRI Kilimanjaro Clinical Research Institute

KIA Kilimanjaro International Airport

KIDH Kibong’oto Infectious Diseases Hospital

MAREMA Manyara Region Miners Association

MoHCDGEC Ministry of Health, Community Development, Gender, Elderly and Children

MUHAS Muhimbili University of Health and Allied Sciences

NIOSH National Institute for Occupational Safety and Health

NTLP National Tuberculosis and Leprosy Programme

OSHA Occupational Safety and Health Authority

PAR Population Attributed Risk

PPE Personal Protective Equipment

PVC Polyvinyl Chloride

RCD Respirable Combustible Dust

REL Recommended Exposure Limit

SADC Southern Africa Development Community

SIR Standardized Incidence Ratio

SMR Standardized Mortality Ratio

SPSS Statistical Package for Social Sciences

SSM Small Scale Miners

TAEC Tanzania Atomic Energy Commission

TB Tuberculosis

TIMS Tuberculosis In the Mining Sector

TLV Threshold Limit Value

TLVTWA Threshold Limit Value – Time Weighted Average

UN United Nations

USA United States of America

VGDF Vapour, Gases, Dust and Fumes

WHC Wits Health Consortium

WHO World Health Organisation

# DEFINITION OF TERMS

Some key definitions as related to occupational health and safety issues include (Reiprich *et al.* n.d.):

- Occupational Health – Refers to health and safety issues at the workplace with a focus on primary prevention of hazards. The ill health among miners has multiple risk factors that may lead to a number of diseases and accidents, including respiratory diseases like Tuberculosis (TB) and silicosis.
- Homogenous Exposure Group – Entails a group of miners with similar exposure to the hazard and monitoring of the sub-group can give important information on the whole group (South African Department of Labour).
- An occupational disease – A disease resulting from exposure to risk factor(s) arising from work (World Health Organisation (WHO))
- Occupational Exposure Limit - Means the time weighted average concentration for an 8-hour workday and a 40-hour work week to which nearly all workers may be repeatedly exposed without adverse health effects (South African Department of Labour)
- Hazard - A danger or risk ((International Labour Organization (ILO))
- Large Scale Mining – Highly mechanized mining operations, mostly operated by transnational mining corporations

*Different countries classify the size of the mine according to the number of staff employed, the size of the yield and the equipment/technology used to mine.*

- Small Scale Miners (SSM) commonly refers to miners who deploy simple and low mechanization mining techniques though the activity itself may be large with respect to number of miners and geographical coverage (Mayala, 2016). Mining activity with a maximum production limit of 50,000 tonnes per year from open cast mining or financing capital of USD 3-5 million (United Nations). A labour intensive mining with low initial capital of 500,000Tsh (Tanzania Mining Policy of 1983). Mining activity that could be termed as ‘*subsistence mining*’ (World Bank). Mining activity that could be termed as ‘*micro scale mining*’ (Southern Africa Development Community Mining Unit). Sometimes the term ‘Artisanal Miners’ is used, referring to ‘illegal’ mining activities. But as in many cases the SSM also have no mining licence or not under the formal sector, they fits both criteria, hence frequently the terms ‘Artisanal Small Scale Miners (ASSM)’ is used.
  - An operational definition as per this study – SSM will refers to mineworkers who are working under the tanzanite mines’ owners or mines’ managers in Mererani and who goes down the mining pits and take part in various mining activities including drilling, blasting and up-lifting of the mine’s rocks.
- Personal Protective Equipment (PPE) – It refers to additional (to the other facility controls and safety systems) protection to workers who are exposed to workplace hazards (World Bank)
- Pneumoconioses - Are a group of interstitial lung diseases caused by the inhalation of certain dusts that leads to tissue’s reaction to the dust. The key cause of the pneumoconioses is workplace exposure. It includes: asbestosis, silicosis, and coal workers’ pneumoconiosis. They results from exposure and inhalation of asbestos fibres, silica dust and coal mine dust (National Institute of Occupational Safety and Health)
- Respirable Crystalline Silica – Is part of the airborne crystalline silica that is capable of entering the gas exchange area of the lungs when inhaled (National Institute of Occupational Safety and Health)
- Silicosis is an incurable lung disease caused by inhalation of dust that contains free crystalline silica (ILO)
- Tuberculosis (TB) – Is a chronic, air born, infectious disease caused by mainly the *Mycobacterium tuberculosis (M.tb)*. An individual become infected following inhalation of infectious droplets from a person with pulmonary TB. The process of acquiring an infection requires sharing of enclosed (poorly ventilated) airspace with another person with the pulmonary form of the disease, commonly a prolonged exposure. Infected individuals with competent immune function are able to contain but not to eliminate the infection ((National Institute of Occupational Safety and Health (NIOSH))

# ABSTRACT

BACKGROUND

The term SSM refers to miners who deploy simple and low mechanization mining techniques, usually of low capital investment. Though the activity may large with respect to number of workers and geographical coverage. It involves exploration, extraction, processing and transportation of minerals. The mining sector in Tanzania is largely accounted by SSM. Because of the risky working environment and almost non-use of PPE, SSM are frequently facing hazards including exposure to respirable crystalline silica and low ventilation.

OBJECTIVE

To determine the mean concentrations of respirable crystalline silica and radon gas within the Mererani tanzanite mining pits, its effects on the miners’ respiratory system and to describe miners’ perceptions and awareness on effects of dust exposure.

METHODOLOGY

The study will utilize a comparison cross-sectional design, with both quantitative and qualitative approaches. The mean concentrations of respirable crystalline silica and radon gas will be measured from randomly selected mining pits. The prevalence of silicosis, impaired lung function and tuberculosis will be determined from the size-based random sample of SSM from the selected mining pits. A purposeful sample of SSM including those with most work experience and those with least work experience will be used to describe the SSM awareness and perceptions of their working environment with respect to their lung health.

USE OF THE RESULTS

Study results will be used as partial fulfilment for a degree of Doctor of Philosophy of Tumaini University Makumira, presented in various academic fora and national/international conferences for sharing scientific knowledge but also for influencing policy changes and enforcement of health and safety occupational regulation.

# CHAPTER ONE

## Introduction

### Background

Globally there are estimated 20 – 30 million individuals directly engaged in small-scale mining, of whom 10 - 50% are women. Relatively more women are found in small scale mining as compared to large scale mining, though most of whom performs other social-economic activities other than the actual mining, including food vendoring (van der Wal and Haan 2010, Eftimie *et al.* 2012, Mayala *et al.* 2016, Ayaaba *et al.* 2017). Some reports have estimated the SSM to account and provide livelihood to around 100 million individuals worldwide. Though many SSM activities are conducted in a primitive and uncoordinated way, it has the chance to positively change the livelihood of many individuals and communities when done in a proper manner (Eftimie *et al.* 2012).

Within the Southern Africa Development Community (SADC) region, the types and levels of mining activities differs between countries, with countries like South Africa (SA) engaging mostly in formal large scale mining with estimated 500,000 workers while Tanzania has mostly the SSM with estimated 1.5 million workers (In Tanzania, the formal large scale mining employs about 15,000 miners). Generally the mining sector account for about 2.5% of the Gross Domestic Product (GDP) (Nelson 2012, Mdee 2015, Tuberculosis In the Mining Sector (TIMS) 2017).

While being significantly preventable, the work related diseases, including those among miners are of important public health concern. A number of occupations, including mining have been associated with exposure to silica dust (more for underground type of mining), sandblasting, construction, cement production, tunnelling, ceramic, pottery and agriculture. Factors determining the exposure risks include size of the dust particle, quartz contents, chemical and physical characteristics (Sharma *et al.* 2016, de Matteis *et al.* 2017, Nakládalová *et al.* 2018). Other exposures have been linked to impairment of lung function, these include pesticides, gases & fumes in occupational settings (Doust *et al.* 2014). The mining sector has been associated with significant burden of work related diseases to both miners and their surrounding communities. Around USD886 million is lost on health as a result of lost human resources, reduced production and training of new working force (Vinnikov 2016, TIMS 2017). The miners are frequently are facing risky environment including heat, noise, vibrations, various forms of dust from silica, wood, coal, cotton and grain, use of crude oil & its associated products and poisonous gases (Bråtveit *et al.* 2003, Skogstad *et al.* 2016). While in one side the small-scale mining has a positive image through creation of jobs and income for families but on the other side the sector has been taken negatively from social, health and environmental side effects. This is more pronounced in regions/areas with limited resources and enforcement of occupational safety and health control measures (Fisher 2007, Sosy 2013, Gottesfeld *et al.* 2015, Ayaaba *et al.* 2017, Sadhra *et al.* 2017).

There is limited accessibility to health services; miners are faced with financial limitation from those with low wages to some who are not being paid at all, and low awareness about the involved risks to ill health. Generally, the situation has been found to be better for middle-income countries as compared to the low-income ones. There are regulations requiring reporting of occupational diseases, but the available reports are unreliable as the data are significantly incomplete, there is no clear and well-functioning mechanisms for reporting the diseases. In addition, most Health Care Providers (HCP) are unaware of key occupational diseases in their locality, making even the little existing information to be highly unreliable. The SADC Charter of Fundamental Rights states; ‘*every worker in the region has the right to health and safety at work and to a healthy and safe environment that sustains human development and access to adequate shelter*’. The ILO Convention No. 155 emphasizes on worker’s rights to services on prevention, detection and compensation of diseases and injuries related to work. The SADC countries approved the ‘*Harmonisation of Mining Policies, Standards, Legislative and Regulatory Framework in Southern Africa*” and a ‘*Harmonisation Implementation Plan*’ that was adopted in 2009. The plan has eight themes and theme number fours addresses safety, health and environmental challenges in the mining sector but to current it is not clear if member states have (and to what extent) implemented the plan (Reiprich *et al.* n.d.). The SADC Declaration (2012) outlines the valuable contribution of the miner into the social economic development while at the same time shows the huge health cost they incur to themselves and their families. The ILO has earmarked the mining sector, especially the SSM as among the main hazardous sectors with main risks including airborne particles, vapours and gases. In an assessment conducted by TIMS, main health risks observed and some presumed diseases among SSM included low/lack of basic health awareness, Human Immunodeficiency Virus (HIV) infection, exposure to dust and lack of protective gears, skin diseases, injuries, overcrowding and low accessibility to health services. Most SSM when fall ill they will opt to go home so as to seek health services through family assistance. This emphasizes on the need to address the currently lack of proper data on the burden and transmission of diseases related to small scale mining as they may be transmitted to communities they return to once they fall sick, hence influencing the burden of diseases in those surrounding communities (International Organization for Migration (IOM) n.d.).

In the African region, SA is the only country that has a clear roadmap towards elimination of silicosis in workplace by 2030. The focus is on prevention of exposure to respirable crystalline silica and the country is implementing the ‘*mandatory code of practice for an occupational health programme on personal exposure to airborne pollutant*’ (Reiprich *et al.* n.d., Gottesfeld *et al.* 2015).

In Tanzania, large-scale miners exist but mostly the mining sector is comprised of SSM. Tanzania’s Occupational Health and Safety Act No 5 of 2003 serves for workers in factories, construction, agriculture, public sector and local government services in mainland Tanzania. Section 96 of this Act enforces any employer with at least 5 employees to have a written policy on health and safety to the workers and to ensure on its effective implementation. The mining Safety, Occupational Health and Environment Protection Regulations of 2010 provide unspecific gas and dust control frameworks. Some of the key health and safety concerns in the mining include TB, silico-tuberculosis, silicosis and Acquired Immunodeficiency Syndrome (AIDS) from HIV. Some countries, including Tanzania have adopted the occupational exposure limits for respirable crystalline silica dust but with exception of SA, others have no guidelines on how to implement these measures (Reiprich *et al.* n.d.). Health detrimental effects of prolonged exposure to respirable crystalline silica (even at levels close to the recommended limits) ranges from silicosis, pulmonary malignancies, renal and autoimmune diseases. Though it may be almost impossible to eliminate silica dust completely, but reduction to safe levels is very feasible (Maciejewska 2015).

Dust is a result, mainly of rock drilling and blasting and is rampant in some occupational areas. Silicon is a common mineral content of the earth’s crust but mostly found in the form of silica (silicon dioxide i.e. SiO_2_, also known as quartz). Silicosis is an ancient incurable inflammatory and fibrotic disease of the lungs, which also disrupt the function of the immune system and other systems/organs of the human body. The disease is potentially preventable by adherence to safety and health regulations at the workplace. Though silicosis has long latency period but it has been reported to occur at young ages, probably as a result child labour and relatively high exposure. The disease is usually classified into three forms: chronic/classic, progressive and acute forms of silicosis. In addition, reports have shown miners exposed to crystalline silica to be at increased risk of lung malignancies and Chronic Obstructive Pulmonary Diseases (COPD). As defined by the American Thoracic Society (ATS) and European Respiratory Society (ERS), the COPDs comprise of disease conditions that are preventable and treatable but leaving the subject with impaired airflow that is only partially reversible. Some reports suggests impairment of lung function to results at silica dust exposure levels of around 0.1 – 0.2mg/m^3^, while silicosis appears to be independent of the cumulative exposure to silica dust. With no silicosis, studies suggesting disabling impairment of lung function to occurs at least after 30 years of exposure to respirable crystalline silica. Some case reports have shown the occurrence of the acute form of silicosis in less than 5 years of exposure to silica dust. The National Institute for Occupational Safety and Health (NIOSH) established a limit of exposure to respirable crystalline silica of 0.05mg/m^3^ for a 10 hours work per day, while the American Conference of Governmental Industrial Hygienists (ACGIH) established a relatively lower limit of 0.025mg/m^3^ – as a mean time-weighted – Threshold Limit Value – Time Weighted Average (TLVTWA). Some reports propose systematic annual spirometry to mineworkers so as to detect any lung function impairment at an early stage and appropriate work re-location (Reiprich *et al.* n.d., Chen *et al.* 2001, 2012, Merget *et al.* 2002, Bråtveit *et al.* 2003, Flynn and Susi 2003, Collins *et al.* 2005, Madl *et al.* 2008, Dahmann *et al.* 2008, Kahuluda *et al.* 2010, van der Wal and Haan 2010, Mohammadyan *et al.* 2013, Fu *et al.* 2013, Gottesfeld *et al.* 2015, HUTYROVÁ *et al.* 2015, Kraïm-Leleu *et al.* 2016, Ngosa and Naidoo 2016, Sen *et al.* 2016, Sharma *et al.* 2016, Vinnikov 2016, Möhner *et al.* 2017, Osman *et al.* 2017, TIMS 2017, Nakládalová *et al.* 2018, Kabir *et al.* 2018). The occurrence of malignancy conditions following exposure to respirable crystalline silica is thought to be the result of electrical charges carried by radon progeny attached with inhaled dust particles. The charged radon progeny are thought of causing mutations in the Dioxyribo-Nucleic Acid (DNA) of the affected cells through radiation effects. Some countries had set their radon levels at working places in the range of 400 to 1000bq/m^3^, 100 to 1000bq/l in drinking water and 200 to 400bq/m^3^ for indoor condition. Those for the Republic of Czech (1000bq/m^3^), Denmark (400bq/m^3^) and Greece (400bq/m^3^) but the International Agency for Research on Cancer (ICRP) had set the level of 500 to 1500bq/m^3^ (Kahuluda *et al.* 2010).

The Global Plan to Eliminate Silicosis elaborates that ‘*today, society possesses all the necessary means to combat this preventable disease and there is no excuse for silicosis persistence throughout the world. In the absence of effective specific treatment of silicosis, the only approach towards the protection of workers health is the control of exposure to silica-containing dusts*’. World Health Organization defines respirable dust as that fraction of inhaled airborne particles that can penetrate beyond the terminal bronchioles into the gas-exchange region of the lungs (Reiprich *et al.* n.d.).

Tuberculosis continues to be among the key global health challenges with Sub Saharan Africa (which is also highly affected by the HIV/AIDS epidemic) carrying the heaviest disease burden. Small-scale miners have relatively high risk of TB as a result of HIV/AIDS and silicosis, while at the same time TB is the main cause of death among individuals with HIV/AIDS, including miners. Tuberculosis transmission is favoured in poorly ventilated, dark and humid environment as is the case in most of the small-scale mining pits in the Sub Saharan Africa. As miners carry a huge TB burden, they also pose high risk to the surrounding communities they come into contact with. The burden of TB among miners in Sub Saharan Africa is estimated to be between 3,000/100,000 – 7,000/100,000 (Reiprich *et al.* n.d., Gottesfeld *et al.* 2015, Ayaaba *et al.* 2017, TIMS 2017), while some countries have reported to have up to 8 to10-times higher burden of TB among miners relative to the general community. In Tanzania, the prevalence of HIV among SSM is about 9%, but generally the data on these diseases in most of the countries is limited (Reiprich *et al.* n.d., Sharma *et al.* 2016, TIMS 2017). The very basic and key health and safety measures are significantly inadequate or completely missing. Noting the high burden of TB in the mining sector and its associated negative effects to the surrounding communities, the Heads of States in the SADC region came up with the ‘*Declaration of TB in the Mining Sector in Southern Africa*’ in 2012. Among other issues, the declaration outlines the high contribution of the mining sector and the miners to the economy and development of the nations in the region but which comes at a huge negative health effects to the individuals miners and their families (Reiprich *et al.* n.d.).

The policy paper on small-scale mining was formulated by the Government of Tanzania in 1983 aiming at transforming the sector into a formal one. This efforts included the establishment of mobile mineral processing plants that were stationed at Buziba (Geita), Kasanga (Chunya) and Kyerwa (Karagwe), one was for gold processing while the other was for tin, but the project failed to materialize (Mwaipopo-ako 1994). By 1999, the number of SSM in Tanzania were estimated to be around 600,000 (Bråtveit *et al.* 2003). The number of SSM has been on increase from estimated 150,000 (1987), 550,000 (1996) to nearly 700,000 (2011) of whom around 27% are women. Estimates of employments in different types of SSM includes: gold - 396,310, building materials - 160,744, gemstones, including tanzanites - 81,710, diamonds - 16,800, salt - 14,321 and copper - 10,500. There is a relatively higher density of SSM in the eastern part of the country as a result of more gemstone deposits along the Usagaran-Mozambique belt, the Usambara and Uluguru mountain ranges and the blue tanzanite at the slopes of Mount Kilimanjaro in Mererani area in Manyara region. With the nation’s vision to become a middle-income country by 2025, key social issues like education and health need to be addressed in an efficient but aggressive manner. This include health of key populations like SSM especially related to diseases of public health importance like TB (United Nations (UN) Women 2016). Though SSM provides significant amount of employments and economic development to the country but most mining activities are unregulated and poses huge safety and health risks to the workers. There are little, if any dust suppression mechanisms and miners are using working tools that are designed for surface mining, hence increasing the risk of disease even more. Also, the miners work in restricted underground spaces with no ear protective equipment, and sometimes they don’t keep enough distance during blasting. This predisposes them to various forms of ear diseases including hearing loss. On the other hand, the high burden of TB among SSM has been linked with overcrowding and low ventilation in the working area. A number of approaches have been in use to determine the extent of work related exposures including ‘single expert vs. panel of experts evaluation’, ‘Job Exposure Matrix’ and ‘self-reported exposure’ (IOM n.d., Mwaipopo-ako 1994, Malisa and Kinabo 2005, Quinlan *et al.* 2009, Boniface *et al.* 2013, Mayala *et al.* 2016, UN Women 2016).

The mining activities are done by making narrow shafts, sometime using locally made hammers and head torches. Blasting is done using low energy explosives while the blasted rocks are brought up using spades, bags/sacks or motor rollers, depending on level of mechanization of a given mine. Most SSM do not use any form of protective gears with respect of inhalation of dust, fumes or toxic gases like sulphur dioxide, nitrous oxide, etc. (IOM n.d., Sosy 2013, Mdee 2015).

In Tanzania, all workplaces are required by law to be registered with Occupational Safety and Health Authority (OSHA), adhere to the prescribed standards and go through the required regular inspections. But the Auditor General report of 2013 showed that just a small proportion of the workplaces were registered with OSHA, which poses a significant challenge in the process of enforcing the health and safety standards. In addition, the Auditors General reports emphasizes that, workplace in the remote rural/peripheral areas are at significant risk of being unregistered completely and this is the case for most SSM like those in Mererani. Occupational Safety and Health Authority has the mandate of conducting occupational health and safety monitoring in all the countries’ workplaces that involve fitness to work assessment on pre-employment and on predetermined regular intervals. Large-scale miners have internal health and safety monitoring mechanisms that meet the British Occupational Hygiene Society (BOHS) level III or IV. The medium scale mines have health and safety services that meet BOHS level I or II. Most of the SSM are not in line with OSHA prescribed standards with no pre-employment health assessment and no regular fitness to work assessment is done (Reiprich *et al.* n.d.).

Tanzania, as it is for other parts of Sub-Saharan Africa in general has significantly high rates of HIV/AIDS, which is among the main risk factors for TB. Miners with significant exposure to respirable crystalline silica, which also in some cases they are faced with poor nutrition and alcohol abuse, have very high risk of getting TB (Gottesfeld *et al.* 2015, Mpagama *et al.* 2015). Tanzania is among the nations visioning for zero new TB infections, zero stigma and zero deaths from TB, HIV and silicosis. The country is also committed to establishment of regular screening of miners and their close contact, referral of those presumed to have TB for laboratory diagnostic services and early initiation of treatment to those found to have TB. As of current, these services are routinely provided by large-scale mining corporations only, hence the urgent need to shift the efforts to the SSM. During the TIMS meeting it was outlined that it is very likely most SSM in Tanzania are primarily dying of silicosis rather than TB (IOM n.d.).

It has been reported that, the tanzanite mineworkers from Mererani accounts for a significant proportion (25%) of patients with re-treatment form of TB and other chronic lung diseases being managed at Kibong’oto Infectious Diseases Hospital (KIDH) and most important, they also account a high proportion of those with poor treatment outcome, including death. Among the characteristics that defines the SSM in Mererani include low awareness about the surrounding health system and health services provided, risky working environment including exposure to silica dust and poor ventilation, low disclosure of illness due to fear of losing their work, hence low access to health services. In addition, as it is for other SSM, the burden of TB is known to be very high among miners in Mererani (Mpagama *et al.* 2015). Miners in Mererani use low tech and old mechanization mostly comprising of worn-out compressors, pneumatic drills, air blowers, jackhammers, generators, hosepipes and low energy explosives. Following completion of drilling and blasting, the rocks are manually put in sacks and carried to the surface on metal containers using simple pulleys. Some SSM uses just light clothe to cover their nose and mouth but most do not use any form of protective gears. Among the major health challenges faced by miners in Mererani, is inhalation of graphitic dust and silica particles during extraction of the gemstone (Malisa and Kinabo 2005, Kahuluda *et al.* 2010, Sosy 2013).

### Literature Review

Most publications on the association between work exposure to respirable dust and occurrence of lung diseases, including COPD comes from personal self-reports of exposures to Vapour, Gases, Dust and Fumes (VGDF), that may be biased depending on one’s perception or knowledge about the situation. Frequently the self-reporting approach has been associated with over estimation of the link between the two. For several years, the effects of work-related exposure of VGDF to COPD has been studied to the extent of suggesting a causal association from respirable pollutants, including dust from silica, wood, coal, grain and also toxic gases, fumes, fibres and mist. Conducting evaluation of work exposure necessitate collection of individual’s data on exposure, of which is lacking in most (if not all of SSM). Work related COPD has been reported to account for about 15% of the total population burden of COPD. Silica (SiO_2_) ranks the second mineral in amount in the earth’s crust and forms the contents of rocks, sand and soil. Given the working environment, mineworkers, smelters, sandblasters, quarrymen, masons and ceramic & glass manufacturers are among the people exposed to silica. The key channel of acquiring silica dust is through the respiratory route. For large scale mining, population-based Job Exposure Matrix (JEM) have commonly being used to monitor exposure patterns, with their coding system mostly depending on professional knowledge of industries, occupational environment and determinant of work exposure. Also some work-based JEM have been established, which monitors the actual occupational data, being it current or historical (Brüske *et al.* 2013, Blanc and Torén 2016, Kraïm-Leleu *et al.* 2016, Ayaaba *et al.* 2017, Möhner *et al.* 2017, Sadhra *et al.* 2017).

A moderate exposure to crystalline silica, of 0.05 – 1mg/m^3^ for a period of 20 to 45 years has been associated with occurrence of chronic silicosis and also other chronic diseases like rheumatoid arthritis. Other diseases that have been correlated with exposure to silica dust include: scleroderma, systemic lupus erythematoses, glomerulonephritis and malignancies of the urinary bladder. The IARC considers silica to have carcinogenic effects in human beings. This is especially key to persons who inhale significant levels of silica causing lung fibrosis. Informative research on dose-effect related to exposure to respirable silica is still a challenge (Steenland *et al.* 2001, Bråtveit *et al.* 2003, Cumberbatch *et al.* 2015, Gottesfeld *et al.* 2015, Möhner *et al.* 2017). Prevalence of silicosis among miners is estimated to around 1% in Zimbabwe while raises up to between 22% - 32% in SA (Nelson 2012, TIMS 2017). Particle size and its concentration in the breathed air are the key factors that drive the pathogenesis of crystalline silica. The respirable crystalline silica particles are those of less than 10uM (PM10) have the ability to enter the tracheobronchial tree and can be captured by the defensive mucus membrane where it can lead to responsive immune reaction and damage to these lung structures. But studies have reported respirable particles of size between 0.5-3uM to be the most fibrogenic among humans. Usually, silicosis will occur 20 years after first exposure, but in situation of heavy exposures the acute or accelerated type of the disease can occur in 1-3 years of exposure. The accelerated silicosis has been reported in gold miners in China, stonemasons in Scotland and garment workers in (Kahuluda *et al.* 2010, Gottesfeld *et al.* 2015).

Silicosis has been associated with increased risk of TB, lung malignancy and heart diseases. Some reports shows there to be estimated 23 million workers exposed to crystalline silica in China and at least 10 million workers in India who are at risk (Bråtveit *et al.* 2003, Fu *et al.* 2013, Ngosa and Naidoo 2016, Möhner *et al.* 2017). As silicosis usually has a long latency period, it requires a well established systematic mechanism of screening the former miners of which is also missing in most cases (Reiprich *et al.* n.d.). In an experimental study on traditional Chinese treatment of silica exposure using ‘kombucha’ among experimental animals (rats), no any clinical abnormalities were noted during the early times of treatment but at the later periods, there was a significant weight loss noted among those exposed to silica. At a one week period following silica injection, the control group was noted to have significantly higher weight (p<0.01) (Fu *et al.* 2013).

In a systematic review and meta-analysis of studies utilizing objective analysis of actual dust exposures in relation to health status of the mineworkers, the high risk of COPD, which is among the global leading preventable causes of ill-health, lung complications and death, was clearly reported (Brueske *et al.* 2014, De Matteis *et al.* 2016, Sadhra *et al.* 2017). Study report from Hong Kong have related work exposure to dust in gemstone mining and processing to occurrence of dust diseases of the lungs, in particular silicosis (van der Wal and Haan 2010), but at the same time some other reports on exposure to mining related asbestos did not show any association with COPD (Ameille *et al.* 2010). Study conducted among mineworkers in Bangladesh report up to 75% of the investigated men mineworkers to have been found with obstructive lung impairment conditions (Kabir *et al.* 2018).

A cohort of at least 2200 white SA gold mineworkers showed incidence cases of silicosis exposure to respirable crystalline silica of concentrations 3μg/m^3^. Other reports from American gold, Chinese tin and black SA gold mineworkers revealed exposures concentrations ranging from 3 – 10μg/m^3^ (Collins *et al.* 2005). Main means of dust control in occupational environment involve application of the wet method and exhaust ventilation, though for respirable quartz, may not provide the necessary reduction of below the ACHIG Threshold Limit Value (TLV) of 0.05mg/m^3^ (Flynn and Susi 2003).

Some reports shows the exposure to respirable crystalline silica in the large mines in China and South Africa to be less than 0.5mg/m^3^, but for SSM in most of the developing world the data are not comprehensive. Report by Dosemeci et al., 1995, cited in another study, showed the exposure to respirable crystalline silica in 20 mines in China to have been lowered from 4.89mg/m^3^ in 1959 to 0.39mg/m^3^ in 1989, similarly following deployment of effective preventive measure during mining activities. Other reports showed the concentration of crystalline silica in gold mines in SA to had a progressive downward trend from 50mg/m^3^ (1905), 2mg/m^3^ (1930), 0.2mg/m^3^ (1938) to 0.05mg/m^3^ (1967), all of these being attributed to significant improvement in ventilation levels and the use of dust reduction approaches during mining. Reports show the levels of free silica in the respirable dust of the gold mines to be around 30%. A report by Watts and Parker, 1995 cited in this study showed the average respirable quartz concentration in the United States of America (USA) underground mines to be less than 0.09mg/m^3^ with a spread of 3.4% to 13.3%. But other reports among tin mineworker in China showed a high proportion (33.7%) to had silicosis and more than 67% of these were diagnosed after quitting the work related exposure (Chen *et al.* 2001, Bråtveit *et al.* 2003). Another study among Chinese minerworkers showed the average exposure to respirable crystalline silica to range between 0.08mg/m^3^ in the group of iron miners to about 0.52mg/m^3^ in the tungsten mineworkers (Chen *et al.* 2012).

The ATS reported the significant increased risk of chronic cough, reduced Forced Expiratory Volume in one second after full inspiration (FEV_1_) and reduced FEV_1_/FVC (Forced Vital Capacity) ratio in relation to exposure to occupational pollutants. The society also estimated the COPD Population Attributed Risk (PAR) to be around 15-20%. But as these estimates are proportions, they depend on how causes other than vapour, dusts, gases, and fumes contribute to the development of COPD. The overall estimate of PAR of COPD due to occupational exposure may be misleading and a more quantitative approach may provide more realistic picture. Most of these reports are from gold and uranium mining activities, but still there is few information related to effects of exposure to poorly soluble low-toxicity particles i.e. biopersistent granular (bg), and especially in tanzanite mining (Brüske *et al.* 2013). The NIOSH established a time-weighted average, single respirable crystalline silica exposure limit of 0.05mg/m^3^, while the ACGIH recommended a much strict TLV of 0.025mg/m^3^ (Gottesfeld *et al.* 2015).

In a systematic search and metaanalysis of peer reviewed article from 1st January 1980 to 13^th^ December 2015 of JEM-based occupational COPD studies published in PubMed and EMBASE between 1980 and 2015, among 29 studies involved in the metaanalysis, based on JEM-based studies, a risk increment of 22% (pooled odds ratio =1.22; 95% CI 1.18–1.27) to COPD was observed for those exposed to workplace air pollutants. In addition, the author reported high risk estimates from general population JEM (expert consensus) as compared to occupational JEM derived from measured data (1.26; 1.20–1.33 vs. 1.14; 1.10–1.19). Self reported exposure to VGDF was noted to have high risk as compared to JEM based exposure to VGDF (1.91; 1.72–2.13 vs. 1.10; 1.06–1.24) and dust, in particular biological dusts (1.33; 1.17–1.51) showed the highest risk estimates to COPD. Common findings from the review included shortness of breath - 5, chronic bronchitis - 15, COPD (diagnosed by physician) – 10 and COPD based on spirometry – 17. Dust was reported to be the frequently reported harmful agent, followed by fumes and fibres. The reports also showed a significantly higher risk (28%) on work related exposure from current (longest done work) as relative to the cumulative exposure risk of 19%. Again, high risk was reported from JEMs general population studies (1.26; 95% CI = 1.20 – 1.33) relative to those from work-based JEM (1.14; CI = 1.10 – 1.19). Results from JEM-based studies showed the dose-effect response and had more modest risk estimation (1.10; CI = 1.06 – 1.24) relative to estimates from self-reported exposures (1.91; CI = 1.72 – 2.13). In addition, the biological dust was shown to have higher risk estimates compared to mineral dust (1.07; CI = 1.05 – 1.09). There was a significant heterogeneity in studies reporting on chronic bronchitis while it was not the case for shortness of breath and the pooled effect for shortness of breath and chronic bronchitis was relatively higher for those on current (longest held work) relative to the cumulative exposure. JEM-based estimates were noted to be reduced relative to the self-reported estimates. But the author reported the finding of publication bias for studies with self-reported physician diagnosed COPD (0.64; p = 0.001, chronic bronchitis (0.36; p = 0.022), spirometry-diagnosed COPD (0.50; p = 0.001 and combined COPD (0.63; p = 0.001) while there was not publication bias on shortness of breath (0.37; p = 0.135), using the Funnel plots and Egger tests. Through meta-regression analysis, the heterogeneity on self-reported physician COPD, were reported to be accounted by gender (coefficient = -0.102; p = 0.046), year of publication (-0.04; p = 0.001) and type of JEM (0.093; p = 0.053) while for chronic bronchitis the accounting factors were the study population (0.256; p = 0.002) and type of JEM (0.256; p = 0.002). Heterogeneity between studies was small though reported to be significant (12 = 46.8%, p = 0.001) that went down to 39.6% when the studies were limited to those diagnosing COPD by spirometry. For studies that utilized self-reported by physician diagnosis, the pooled risk estimate was found to be significantly higher (1.36; 95% CI = 1.26 – 1.47, p = 0.001) relative to COPD reported from spirometry (1.16; 95% CI = 1.12 – 1.20), making an overall odd increase of 22% (95% CI = 18 – 27%) across the studies. Women were reported to show higher risk of COPD compared to men, findings from population-based studies relative to occupational-based studies and also findings from case-control studies relative to other study designs. The systemic review found a significant heterogeneity between the studies (I2=46.8%, p = 0.001) that if limited to studies that used spirometry to determine the COPD, it comes down to 39.6%. Pooled risk was significantly higher (p = 0,001) for the studies utilized self reported physician diagnosis (1.36; 95% CI 1.26 – 1.47) as compared to those utilizing spirometry (1.16; 95% CI 1.12 – 1.20), this leads to about 22% raise in odds (95% CI 18% - 27%) among all the studies. Findings also showed COPD risk was higher in women relative to men, general population based studies as compare to occupational based and in case-control designs relative to other designs. Also, studies that used expert general population JEM had relatively higher risks (1.26; 1.20–1.33) versus the work based JEM (1.14; 1.10–1.19) from measured exposure data. Studies utilizing JEMs reviled a dose-response effect and also gave more modest estimates (1.10; 1.06–1.24) relative to self reported exposures to VGDF (1.91; 1.72–2.13). The biological dusts produced higher risk estimates (1.33; 1.17–1.51) compared to mineral dusts (1.07; 1.05–1.09). The review reported significant heterogeneity in studies reporting on chronic bronchitis, though not for shortness of breath and the pooled effect size on shortness of breath and bronchitis was higher for the current workers relative to the cumulative exposure. Studies using JEMs showed low risk estimates relative to the self reported exposures. Reports on chronic bronchitis from occupational based JEMs had higher pool estimates (1.73; 1.37–2.19) relative to those utilized population based JEM (1.29; 1.23–1.35). However, the review reported the likely publication bias for self reported physician diagnosed COPD (bias = 0.64, p = 0.001), chronic bron­chitis (bias = 0.36, p = 0.022), spirometry diagnosed COPD (bias = 0.50, p = 0.001) and combined COPD (bias = 0.63, p = 0.001) as outcomes. However, the plots suggested no publication bias for breathlessness (bias = 0.37, p = 0.135). Reported factors that influenced heterogeneity in self reported physician diagnosis of COPD included: gender (coefficient = -0.102, p = 0.046), publication year (-0.040, P = 0.001), and JEM types (0.093, p = 0.053) and for chronic bronchitis was study population (0.256, p = 0.002) and JEM types (0.256, p = 0.002) (Sadhra *et al.* 2017).

In a systematic review and meta-analysis that search publications in PUBMED and EMBASE in which 55 studies were included, of them 27 were used in evaluating the effect of work exposure to bg dust (though not all 27 could be included in meta-analysis), Part of the review report showed that some studies in USA and Jordan reported no impaired lung function among cement workers following occupational exposure to dust while other studies from Tanzania and Iran had resulted into a significant association of the same workers. Reports from workers in metal industries like iron, aluminium and steel showed contradicting results in terms of exposure effect. The review of longitudinal studies showed varied effects on lung function among mineworkers exposed to dust, though also the patterns of exposure also varied extensively in terms of dust type, composition and level of exposure. Those working in large open pit bauxite mines (Australia) had low exposure with no impaired lung function while those working in underground manganese mines in Iran and potash mines in Germany showed the opposite, with significantly reduced FEV_1_ and FVC among the exposed workers. In this review of cross-sectional studies with similar endpoints, the mean FEV_1_ of mineworkers exposed to bg dust was 160mls (95% CI: 40-270 ml) lower with respect to the unexposed mineworkers, which in percentage predicted it was 5.7% (95% CI: 2.71-8.62%) less for exposed workers. In addition, when the standardized mean difference was considered, again it showed reduced FEV_1_. The mean difference of FEV_1_/FVC between the bg dust exposed and unexposed workers showed a significantly lower level among the unexposed -0.25 (95% CI:-0.09 to -0.41). The meta-analysis of the longitudinal studies showed the mean annual decrease in FEV_1_ of 6.3mls higher among the bg dust exposed workers relative to the unexposed ones. Using reports that evaluated cumulative effects (mg/m^3^/year), the meta-analysis reported reduction in FEV_1_ of 1.6ml/mg/m^3^/years, meta-analysis 1.58ml (95% CI: 1.24-1.93ml). Through the Global Initiative for Chronic Obstructive Lung Disease (GOLD) criteria, two studies showed a raised odds ratio of COPD (FEV_1_/FVC< 70%), one study reporting 1.06 and the other study 1.07, which is equivalent to a raise of about 1mg/m^3^ bg dust (Brüske *et al.* 2013).

In assessing exposure to respirable crystalline silica, key uncertainties have to be taken into account to avoid bias estimates, these include mineral forms of silica, the activities that lead to dust generation, the use and types of protective equipment used, assessment of the respirable dust part and the quartz contents. In several studies, the quartz contents are not being measured objectively but rather just calculated (Dahmann *et al.* 2008).

In a review of studies in PUBMED in 2009 on exposure to respirable quartz and associated diseases in agricultural sector in SA, the review that involved 17 studies showed the presence of significant risk to respirable quartz on farm soil though didn’t show an association with disease occurrence. The measured respirable quartz ranged from undetectable levels to 626μ/m^3^ (during wheat planting) but in most, the levels of respirable dust were less than the recommended work limits of 2mg/m^3^. Of the 138 respirable dust samples, 12 (9%) samples and 18 (13%) samples had above the work exposure limits of 2mg/m^3^ and 100μ/m^3^ for respirable dust and quartz contents respectively. Among the respirable quartz samples, 57% were above the ACGIH TLV of 25μ/m^3^. The quartz content in the respirable dust ranged between 0.3 – 94.4%, median of 13.4% (Swanepoel *et al.* 2010).

In a study on pooled exposure-effect involving 10 silica exposed cohorts that combined more than 65,000 workers, the log of cumulative exposure with a 15year lag was found to be strong predictor of pulmonary malignancy (p = 0.0001) and the findings were consistent across studies (heterogeneity analysis, p = 0.34). The lifetime (age 75) estimated excess risk of pulmonary malignancy for exposure to respirable crystalline silica between the age of 20 – 65 at the level of 0.1mg/m^3^ was reported to be 1.1 – 1.7% above the baseline risk of 3 – 6%, which strongly concur with IARC to group work related respirable silica as carcinogen (Steenland *et al.* 2001).

The incidence of silicosis of severity 1/1 or greater show lifetime risks of 55–92%, assuming 45years of continuous exposure to silica at 0.10mg/m^3^, which corresponds to a cumulative exposure of 4.5mg/m^3^/year (Bråtveit *et al.* 2003). Exposure to respirable crystalline silica has been reported to be highly harmful as the phagocytised silica granules in the alveoli leads to fibrosis and lastly silicosis (Brueske *et al.* 2014).

Reports on dust related diseases, and especially silicosis among mineworkers in Tanzania is still scarce. In a review to assess the publications and report on TB, HIV and silicosis among mining communities in Southern Africa, most 47% were from SA, while Tanzania, Malawi and Lesotho combined accounted for 7% (and even on this group, those reporting on silicosis and other dust disease were very less). The review showed the prevalence of TB and HIV in Southern African countries to be 9% and 47% respectively (TIMS 2017).

In a study that evaluated respiratory diseases among gold mineworkers in Ghana, a significant association between age, educational status, marital status and drinking alcohol and presence of respiratory diseases was reported. Occupational respiratory diseases are associated with six-times mortality rates as compared to occupational accidents, and have been linked to major social economic setbacks within the community. The extent and severity of the pulmonary disease is associated with duration and density of exposure and also the types and size of the particles a person is exposed to. The ILO estimates shows work related mortality of 6,300 within 24 hours of which 5,500 death are directly from occupational diseases, also estimates shows the existence of 160 million events of non-fatal occupational related diseases each year (Ayaaba *et al.* 2017).

A report of a study from black South African gold mineworkers showed respiratory disorders related to silicosis to be among the main diseases within this population and had an association with the duration of underground working. In USA, coal mineworkers employed before 1970 are reported to have high prevalence of chronic bronchitis (35%), shortness of breath (43%) and wheezing (42%) as per the National Study of Coal Workers’ Pneumoconiosis. Seixas et al., reported a prevalence of 28% for cough, 21% for chronic bronchitis, 22% for shortness of breath and 27% for wheezing. The common respiratory symptoms reported were cough, chest pain (25.4%), wheezing (21.2%) and shortness of breath (10.6%). At least 85% of the workers perceived the symptoms to be occupational related and the common attributed factor was dust exposure (64.1%). Commonest respiratory diseases encountered were asthma (37.5%), pneumonia (14.3%) and bronchitis (9.7%). In this study, among those responded through a questionnaire, 77.3% were Christians, at least 80% were married, around 47% reported to had Junior Secondary School education, 7.5% had Senior Secondary Education and 8.4% had Tertiary Education. Around 87.7% were Ghanians, about 2.2% reported to smoke cigarettes and at least 50% were alcohol drinkers. The mean (sd) duration of working in mining was 13.1 years (6.8), ranging from 1-47years. The mean (sd) duration of work per day was 12 hours (0.6), ranging from 2-14hours. Around 93.4% were exposed to dust and 89.4% were exposed to beyond limits (high/low) temperature in their working environment. The study found a significant association between age and the studied respiratory diseases. Of the workers with emphysema and pneumonia, significant proportion was between 30-40 years of age while bronchitis was highest to those above 50years of age. In addition, there was a significant association between the level of education and all the reported respiratory diseases; proportion of those with emphysema and pneumonia was lower among workers with tertiary and diploma education levels. The association between smoking and respiratory diseases was not significant. The work duration was noted to have an association with asthma (p = 0.027) and pneumonia (p<0.001). Asthma was reported to have higher proportion to those with work duration of 5-10years (52.2%) and was lower among those worked between 10-15years (25.9%). Still several countries have no a systematic mechanisms of collecting and storing data related to occupational disease, which are vital in establishing effective preventive approaches. Some other reports have strongly suggestion a reciprocal association between Social Economic Status (SES), mostly measured in terms of income & level of education and occurrence of COPD (15, 45).

Exposure to dust was also reported to be associated with pneumonia (p<0.001) and emphysema (p<0.001) but was reported not to be associated with asthma and silicosis. Proportion of mineworkers with pneumonia was significantly higher to those having 10-20 years of dust exposure (21.9%) and was significantly lower to those exposed to dust for less than 10years. Generally, proportion of mineworkers with respiratory diseases was higher to those exposed to dust as compared to the unexposed ones. Occupational hygiene, especially the place of bathing after work has a significant association with respiratory diseases as the proportion of emphysema and pneumonia reported to be significantly higher among those taking bath a the workplace relative to those taking bath at home. Also age was noted to be associated with high risk of asthma, pneumonia and emphysema, a one-year extra of age was associated with raised risk of asthma (OR; 95% CI = 1.12; 1.06-1.19) while for emphysema, an inverse association was noted with a 5% less of risk for one year raise in age (OR; 95% CI = 0.95; 0.92-0.98). Tertiary level of education was associated with significant decrease of the odds of pneumonia as compared to those with junior secondary education, also marital condition of men workers has a significant association with emphysema in two different models (OR; 95% CI = 0.60; 0.37–0.85) and model 2 (OR; 95%CI = 0.53; 0.34–0.82). Drinking alcohol had raised odds with asthma and pneumonia and lowered odds of emphysema. Long duration of mining was related to increased risk of pneumonia (OR; 95% CI = 1.04; 1.00–1.07) and emphysema (OR; 95%CI = 1.04; 1.01–1.06). Extreme temperature had a significant higher risk of pneumonia (OR; 95% CI = 4.62; 1.62–12.96) (Ayaaba *et al.* 2017).

In a review aiming at studies that determine the association between work related dust exposure and COPD, the search on MEDLINE from January 1966 to July 1991, personal communication with at least 30 international experts and SCISEARCH among others, there was a significant association between the cumulative exposure to respirable dust and impairment of lung function in all the studies. The burden was estimated at 80 (95% CI; 34 - 137) among 1,000 coal mineworkers (non-smokers) with a cumulative dust exposure of 122.5gh/m^3^ (equivalent to around 35years of work under mean respirable dust exposure of 2mg/m^3^) expected to end with clinically significant (more than 20%) reduction on FEV_1_ that is attributable to dust exposure. For smokers; among 1,000 mineworkers the estimates were 66 (95% CI; 49 - 84). For non-smokers gold miners; the clinically significant impairment of lung function attributable to dust exposure was around 3-times higher compared to coal mineworkers at one-fifth less the cumulative exposure to respirable dust (21.3gh/m^3^), the highest exposure observed among gold miners. Generally the study reported the work related exposure to respirable dust as a key cause of COPD while the risk to be significantly higher for gold, relative to coal mineworkers (Oxman *et al.* 1993). A systematic review in PUBMED and EMBASE from 1970 to 2010 and meta-analysis on occupational exposure to respirable quartz and COPD through impairment of the FEV_1_ and the FEV_1_/FVC. Of the 55 studies analysed, 11 showed the presence of an association between exposure to respirable quartz and COPD. Most studies reported a negative association between work exposure to crystalline quartz and FEV_1_ and FEV_1_/FVC. From meta-analysis of the cross-sectional studies, the mean FEV_1_/FVC was reported to be lower and FEV_1_ among those exposed was 4.6% lower as compared to those not/with low exposure. Work exposure to respirable quartz showed a significant reduction on FEV_1_ and FEV_1_/FVC, features consistent with COPD (Brueske *et al.* 2014). A systematic review and meta-analysis of population-based studies utilizing JEM to determine the association between exposure to respirable dust and COPD reported low exposure to mineral dust and high exposure to gases/fumes to have an association with high risk of COPD. Both low and high exposures to biological and mineral dust were reported to have significant association with chronic bronchitis. The author reported the expert opinion to be presence of significant association between work related exposure (assessed through JEM) and risk of both COPD and chronic bronchitis (Alif *et al.* 2016). The chronic impairment of lung functions is presumed to be predictors of both morbidity and mortality. In a review to assess the impact of exposure to organic dust on impairment of lung function, of the 20 studies assessed, 14 were found to show an association between exposure to organic dust and impairment of lung function, though the findings were inconsistent with no link with specific work exposure. The meta-analysis showed a significant difference in decrease of FEV_1_ for the exposed relative to the unexposed, 4.92ml/year (95% CI; 0.14 – 9.69) while there was no significant change with respect to FVC. In addition, 12 studies reported a significant exposure-effect association between organic dust and lung function. But the findings were generally not consistent along various study designs (Bolund *et al.* 2017). Some study reports have attributed about 20% of all prevalent events of COPD to work related exposure to VGDF. In a systematic review from EMBASE and PUBMED from January 1990 to 31 August 2016 aiming at assessing studies that reported on risk estimates of COPD morbidity/mortality or spirometry-based airway obstruction among construction workers, 9 of the involved 12 studies showed a significant association between the construction occupation and occurrence of COPD (Borup *et al.* 2017).

Various studies have shown the association between work related exposure to both organic and inorganic dust to chronic bronchitis and reduced FEV_1_, with an additive effect on smoking. In addition, for coal workers it was found young workers to face more impairment of lung function as compared to older workers. The reports suggest the extent of occupational impairment of lung function to be less relative to that seen as a results of smoking (Garshick *et al.* 1996). There has been consistent reports from population-based studies showing an estimated 15% of the COPD burden to be associated with occupational exposures of which are potentially preventable. From a systematic review on prevention, identification and management of work related COPD, it was reported that minimizing work related exposures to VGDF could be the most effective approach in controlling COPD recommend yearly monitoring of lung function among workers so as to identify those with rapid impairment of lung function (Fishwick *et al.* 2015). Report from a systematic review and meta-analysis of related studies from PUBMED, MEDLINE, EMBASE and Web of Science from October 2012 to May 2014, showed a raised incidence of BC in 42 out of 61 work types and also raised of BC-specific deaths in 16 out of 40 work types, while lowered incidence and deaths were noted in 6 out of 61 and 2 out of 40 work types respectively. The risk was noted to be greater in men with standardized incidence ratio of 1.03 (95% CI; 1.02 – 1.03; p<0.001) (Cumberbatch *et al.* 2015).

Systematic review of cohort and case-control studies and meta-analysis reported a significant raised risk of cancer of the larynx (pooled OR = 1.39, 95% CI: 1.17-1.67) among those exposed to silica dust, following adjustment for smoking and alcohol use, from the case-control studies. Though not statistically significant, but also there was noted an increased risk from the cohort studies, pooled SMR of 1.38 (95% CI: 0.79-1.96) for patients with silicosis and a pooled Standardized Mortality Ratio (SMR) of 1.13 (95% CI: 0.82-1.45) and a pooled Standardized Incidence Ratio (SIR) of 1.50 (95% CI: 0.59-2.42) among those exposed to silica dust (Chen 2012).

There are several report on dust exposures to mineworkers, but most are limited to middle to large scale mining and mostly comes for investigative reasons, in addition most of the report are from gold mining operations while tanzanite mining as a completely different form of mineral, may be associated to different levels of silica contents (Bråtveit *et al.* 2003).

A study report from Zambia showed the burden of TB among underground mineworkers to be around 9.5% (Ngosa and Naidoo 2016)(52). In Tanzania, the country’s TB incidence is estimated at 327/100,000. HIV prevalence among SSM is around 8.9%. Prevalence of silicosis among mineworkers is around 1.6% and reports shows a significant amount of SSM being constantly exposed to silica dust. Most SSM mines are located in communities with low social economic levels associated with low awareness and access on diseases and health services, making health related data fro these areas to be a challenge. SSM are relatively at risk in relation to respirable dust, accidents, hearing impairment, etc. Among the key challenges facing the mineworkers, in particular the SSM include limited published data on safety and health, including diseases related to exposure to dust (Malisa and Kinabo 2005, TIMS 2017). As it has been noted with other SSM in the region, most SSM in Tanzania conduct mining activities without dust control measures in place (Gottesfeld *et al.* 2015).

The evaluation found that there the awareness and knowledge on lung diseases related to the mining occupation is very low hence even its reporting does not exist and during evaluation there was just a single occupational health physician (retired), who had been certified to make diagnosis of pneumoconiosis (Reiprich *et al.* n.d.).

The study conducted in sampled villages in Tanzania were gold is being mined, utilized battery-operated sampling pumps in which samples were collected so as to assess the type and concentration of crystalline silica contents of the ore from the mineworkers breathing zone. The samples were analysed by X-ray diffraction at an accredited laboratory. Exposures to crystalline silica were found to be above the required exposure limits of 16.85mg/m^3^ in the underground drilling, which is 337-times higher than the Recommended Exposure Limit (REL) as per the United States NAOSH. This higher exposure figures raised the risk of silicosis, which also increases the one’s risk of suffering TB. Studies have report the prevalence of TB among miners to be 5-6 times higher, but others up to 15 time higher than that in the general community. The miners also increase the burden of TB in the general population by infecting others while they visit their families. In an investigational report to evaluate exposure to respirable crystalline silica in Artisanal Small-scale Gold Miners (ASGM) in Tanzania showed 97% of the 32 air samples had exposure levels above the NIOSH REL, with their average being 4-times higher the NIOSH REL. The average density of respirable crystalline silica for above ground activities was 0.19mg/m^3^. The average exposure during below ground drilling was 16.85mg/m^3^ which was 337-times higher than the NIOSH REL. The main form of crystalline silica found in the bulk ore samples was quartz of which its density span from 28.7% (sample below crusher) to 67.9% (at the base of the mining shaft), and no cristobalite or tridymite were found (Gottesfeld *et al.* 2015).

The reports showed the median quartz and graphite amount of the respirable and total dust to be 13.6% and 5.5%, respectively. During drilling, blasting and shovelling are done, the exposures were 15.5mg/m^3^ (total respirable dust), 2.4mg/m^3^ (respirable quartz), 1.5mg/m^3^ (respirable graphite) and 28. 4mg/m^3^ (total dust). If only shovelling and loading in the sacks was done, the median exposures were 4.3 mg/m^3^ (overall respirable dust) and 1.1mg/m^3^ (quartz) (Malisa and Kinabo 2005). Another study documented high levels of respiratory diseases among coal miners in Tanzania and another report also in Tanzania showed relatively high prevalence of respiratory related symptoms among the evaluated mineworker: dry cough – 45.7% and shortness of breath – 34.8% (Ayaaba *et al.* 2017).

In Mererani tanzanite mines, there is significantly high exposure to respirable dust during the processes of drilling, blasting and shovelling to about 15.5mg/m^3^ in which the respirable quartz account to 2.4mg/m^3^ and graphite to 1.5mg/m^3^. The total amount of dust goes up to 28.4mg/m^3^. Air supply is done using compressors ranging from small to medium level ones but in most cases the supplied air is insufficient and the consistency is unreliable, some have reported occasions of purposeful cutting off the air supply to the underground tunnels. Aside from other ill-health conditions, the poor and unregulated air supply lead to poor ventilation where the miners are working, and this is among the key factor that increases TB transmission (Malisa and Kinabo 2005). A pilot study done in August 2001 aimed to evaluate level of dust exposure during working and assess its association with chronic lung diseases among mineworkers in Mererani. It involved personal sampling of respirable dust – 15 and total dust – 5 done in a three consecutive days in a mine with a total of 50 workers, showed the median crystalline silica content and the combustible content of the respirable dust samples to be 14.2% and 5.5% respectively. The assessed mine had a day shift of 5-8hours. During drilling, blasting and shovelling, the median levels of respirable dust were 15.5mg/m^3^ that of respirable crystalline silica was 2.4mg/m^3^, respirable combustible dust was 1.5mg/m^3^ and total dust was 28.4mg/m^3^. But the median exposure levels for respirable dust was 4.3mg/m^3^ and that of respirable crystalline silica was 1.1mg/m^3^ when just shovelling and loading of sacks were done. Generally the study reported the exposure to respirable crystalline silica to be relatively high during underground mining activities and mineworker has high risk of suffering silicosis. In Mererani, there are around 300 mine firms with an estimated 15,000 mineworkers. Among all these, the study (pilot) selected just one mine with just 50 mineworkers to conduct the assessment. The study also reported the working environment in the assessed mine to be very risky in terms of narrow unsupported shafts, failure to use the wet method of suppressing dust generation during drilling and blasting, using of primitive low mechanized tool and failure to use any form of PPE like respirator. Though the concentration may differ from point to point and on depth of mining shaft, this pilot study report showed the exposure to respirable dust and crystalline silica among SSM in Mererani to be very high as compared to those documented in SA, China and USA. The personal dust assessment was done to 21 mineworkers with a mean working duration of 4.9years. For the first two days (drilling and blasting) the median exposures of respirable dust was 15.5mg/m^3^, respirable crystalline silica was 2.4mg/m^3^ and total dust was 28.4mg/m^3^, the maximum level of crystalline silica recorded was 3.4mg/m^3^, also the median exposure to Respirable Combustible Dust (RCD) was noted to be high at 1.5mg/m^3^. For the third day that involved shovelling and loading of the rocks in the sacks, the median exposure to respirable dust was 4.3mg/m^3^, that was significantly less as compared to that in first and second days (p = 0.002; Mann–Whitney non-parametric test). This was a similar picture with crystalline silica, RCD & total dust (though the author commented that the statistical significance were not tested due to small samples). In this study, the three days pooled data showed the combined median exposure to respirable dust to be 10.6mg/m^3^ while the median exposure to respirable crystalline silica to be 1.4mg/m^3^. The median crystalline silica for the 9 samples was 14.2% with interquartile range 10.5%-16.4%, and RCD for the 6 samples was 5.5% with interquartile range of 2.1%-20.8%. In addition, for the total dust samples the median contents of calcium was 7.4% (IQR 4.7-9.2%), aluminium – 3.9% (IQR 2.7-4.7% and iron – 1.6% (IQR 0.7-2.2%). The median exposure to calcium was 1.1mg/m^3^, which is equivalent to 1.5mg/m3 calcium oxide. The median exposures to the other elements were <1.0 mg/m^3^, and <10% of their respective TLVs. The exposures to arsenic, silver, cadmium and lead were <1.0 μg/m^3^. In general the pilot study reported very high levels of dust exposure during the underground mining activities that was pronounced during drilling and blasting activities which was 50-times higher compared to the TLV limits. The median level of crystalline silica was reported to be 14.2%. Though the contents of respirable dust was low during shovelling and loading of rocks but the levels of respirable crystalline silica were reported to be very high relative to the respective TLV. As currently reported (personal communication), that still the use of protective gears like respirators in non-existent, it is expected the changes in exposures that mineworkers are facing currently to be, mainly, the results of changes in the rock structure due to changing in the depth of mining shafts, from the estimated 300-400m in late 1990s to the current estimates of 800-1000m. But again, since this estimates are just from pilot study, the current study is expected to come up with more realistic estimates of the real exposures that mineworkers are facing. The author cited reports from Hnizdo and Sluis-Cremer, 1993; Steenland and Brown, 1995; Kreiss and Zhen, 1996; Chen et al., 2001, that showed the mean time from the initial exposure to the commencement of silicosis to be 18-41years while from the author’s pilot study in Mererani the mean number of occupation for the 21 mineworkers in the underground mines was 4.9years, taking the median exposure to crystalline silica during the three days of study as 1.4mg/m^3^, it gives a cumulative exposure of 6.9mg/m^3^-yr (Bråtveit *et al.* 2003).

Usually there are 100 – 120 minewokers in one shaft per shift, while small compressors with capacity of 11.4m^3^/min are being utilized for both drilling and air supply, which was reported to be insufficient. At least 90% of all mining operations in Mererani do not use any dust reduction mechanisms (wet drilling) and just about 10% have been reported to where some form of face masks. In a study aimed at evaluating the impacts of ventilation systems and air pollution among tanzanite miners, the mean of Carbon Monoxide (CO) concentration was found to be 66.2ppm (highest of 102ppm) that was 2.5-times higher compared to the upper limit recommended by Ontario Ministry of Labour. Again the mean concentration of respirable dust was 8mg/m3 that was 4-times higher to the recommended limit (Mayala *et al.* 2016). Another assessment done in Kiwira Coal Mine by Mamuya (2006), documented the densities of the respirable dust of 10.30mg/m^3^ and quartz of 1. 28mg/m^3^ both of which are high relative to the set limit of 2mg/m^3^. A study done to evaluate the concentration of respirable dust among SSM in Mererani (Bråtveit *et al.* 2003) showed the overall median respirable dust concentration of 10.6mg/m^3^, the median concentration of respirable crystalline silica of 1.4mg/m^3^. Again in Mererani tanzanite mines, (Malisa and Kinabo 2005) reported the density of respirable dust to be 15.5mg/m^3^, that of respirable quartz to be 2.4mg/m^3^ and of respirable graphite to be 1.5mg/m^3^, all of these occurs during the activities of drilling, blasting and shovelling. The density of total dust was reported to be 28.4mg/m^3^. The current study came to assess the concentration of radon gas and respirable ore dust in the underground mining shaft of Mererani. As these studies assessed respirable dust density for just three to four days, and were done about 20 years back, when the shift were estimated to be around 300m in depth, while currently the depth of most shaft is between 800 to 100m, it is very likely the exposure patterns have significantly changed (Kahuluda *et al.* 2010).

The study report many mineworkers to have silicosis and about 6.6% of all miners having TB and of all the accidents reported in Mererani mines between 2005 to 2014, suffocation accounted for 29% (Mayala *et al.* 2016).

The study was conducted to estimate the density of radon gas and respirable dust in Mererani tanzanite mines. The radon gas density was measured in terms of disintegration per second per cubic meter and ranged from 40.1bq/m^3^ to 4.2x103bq/m^3^ with the geometric mean of 118.4bq/m^3^ which is below the International Commission on Radiological Protection (ICRP) workplace guidance level of 500 – 1500bq/m^3^. Estimated average effective dose per year was noted to be 1.6mSv that is significantly less than the external exposure effective dose per year of 20mSv and the organ dose limit per year of 2.4mSv (Kahuluda *et al.* 2010).

*Literature Search Strategy*

Key words (and their synonyms) related to the main and specific objectives, their narrower and broader terms and Boolean operators were used to search the relevant references from electronic sources. The search sources included the Google Scholar and PubMed/Medline. Among the key words used included: ‘mineworkers AND exposures’, ‘mining AND dust’, ‘radon AND exposure’, ‘silicotuberculosis’, ‘silicosis’, ‘pneumoconiosis’, ‘Mererani’, ‘Mirerani’, ‘tanzanite AND health’, ‘miners AND health’ and ‘miners AND awareness’. Also the HINARI search engine using specific subjects and journals related to the topic of study was used to find appropriate references.

Other references were obtained as hard data from KIDH patients’ registers, NTLP reports and policy guidelines and also from previous PhD and MSc dissertations.

### Statement of the problem

Most current and former tanzanite miners in Mererani have been attending KIDH seeking health services as a result of signs/symptoms related to lung diseases for more than 35 years (from available hospital registers). Almost all miners visiting KIDH are found to have one or a combination of TB, chest radiological findings suggestive of silicosis and/or lung malignancies but also some are found to have impaired lung functions with both obstructive and restrictive findings. At KIDH 95% i.e. 95,000/100,000 miners with chest radiological findings suggestive of silicosis dies within 1 to 3 years of making the findings (whether or not the miner has TB). This strongly suggesting the occurrence of a highly progressive form of silicosis among the tanzanite miners. Though being an incurable disease, silicosis is significantly preventable through adherence to work health measures including the use of PPE. Most available data on exposure to respirable crystalline silica and its effects among SSM comes from gold mining and most are from outside Tanzania, including South Africa, China and Latin America. These data shows the development of silicosis in most of the mineworkers to take a long duration of about 20 to 40 years. This does not relate to observational clinical data among tanzanite SSM from Mererani with chest radiological features suggestive of silicosis who report exposure duration of around 5 to 15 years. One of the explanation for this finding could be presence of completely different concentration of silica in the respirable dust of the tanzanite mines but also differences in miner’s working environment. This could have an effect on the occurrence of TB among the SSM as silicosis also impairs the functioning of the body’s immune system which is key in the prevention of TB disease. On the contrary, 90% of miners found to have TB with no any chest radiological findings suggestive of silicosis or lung function impairment get cured of TB though about 30% of these will remain (after completion of TB treatment) with one or more symptoms related to complications of TB.

Little is known about the concentrations of radon gas in the pits of Mererani tanzanite mines, a gas that has been associated with occurrence of lung cancers. Its worthy investigating the presence and concentration of radon gas as some SSM attending KIDH have been noted to have clinical and chest radiological findings suggestive of pulmonary malignancies.

The tanzanite gemstone is found and mined only in a small localized area of Mererani in Simanjiro District, Tanzania. As a result, the silica contents of the rocks and concentrations of radon gas in the mining pits may significantly differ from those in the gold and other mineral mines from which most of the report comes from. In addition, the available information on dust contents in Mererani tanzanite mines is about 20 years old, during when the depth of most pits in Mererani were around 200m while currently most pits goes to a depth of 800-1000m. This may have association with changes in the silica contents of the rocks and radon gas emissions from the rocks.

Little is known, from clinical work, about the miners’ perceptions and awareness on effects of exposure to respirable crystalline silica and radon gas. Information from clinical work suggest most SSM to little awareness on effects of exposure to respirable dust (completely not aware of the silica content of the dust) and some perceive the exposure to the mining dust, which is usually associated with blackish discolouration of the skin as a implication of becoming rich in the future.

### Study Justification

The study will provide important and current information on the extent of exposures to respirable crystalline silica and radon gas among the miners in Mererani. In addition, the study will provide key information on the association between these exposures and the occurrence of silicosis, impaired lung functions and TB among the miners.

The reports generated from this study will assist in ensuring effective and sustainable enforcement of health and safety regulations among the SSM through educational and sensitization programs but also through advocacy to government authorities. This will allow the miners to lead a healthier work environment and be more economically productive. In addition, showing the association of exposure to respirable crystalline silica and/or radon gas with occurrence of silicosis, TB and/or lung function impairment will set ground other current mineworkers with similar work related diseases (and legal dependants of miners who have died of work related diseases) to be considered for compensation.

### Study hypotheses

**Null Hypothesis**

Tanzanite SSM in Mererani are not exposed to beyond the allowable limit concentrations of respirable crystalline silica and radon gas and do not experience any ill-health events related to these exposures.

# CHAPTER TWO

## Objectives

### Broad objective

To determine the mean concentrations of respirable crystalline silica and radon gas in Mererani mines, its effects on the miners’ respiratory system and to describe the perceptions and awareness of the miner on the effects of exposure to dust.

### Specific objectives

1. To determine the mean concentration of respirable crystalline silica among tanzanite mining pits in Mererani.
2. To determine the mean concentration of radon gas within the mining pits of the Mererani tanzanite mines.
3. To determine the proportion of SSM with silicosis among tanzanite SSM in Mererani.
4. To determine the proportion of SSM with impaired lung function among tanzanite SSM in Mererani.
5. To determine the proportion of SSM with tuberculosis among tanzanite SSM in Mererani mines.
6. To describe the perceptions and awareness of tanzanite miners in Mererani on the effects of exposure to dust.

# CHAPTER THREE

## Methodology

### Study design

A comparison cross sectional design utilizing both quantitative and qualitative methods will be used, by determining the mean concentrations of respirable crystalline silica and radon gas during working hours from the sampled mining pits and also to determine the presence of silicosis, impaired lung function and/or TB among the selected mineworkers. Comparison group will be sampled from communities in areas around the Mererani mines located within a distance of around 50kilometers from the mines’ fence. Such design has been chosen as it will satisfy the analysis of the association between work related exposures and occurrence of the lung diseases of interest. But also using other designs would be limited by time as both disease of interest usually have long latency period.

### Study site

The study will be done at the tanzanite mines, located in Mererani (Mirerani). Mererani (sometime named Mirerani) is located in Simanjiro District, in Manyara Region in northern part of Tanzania bordering Kilimanjaro Region (northeast), Arusha region (north), Tanga (east), Morogoro and Dodoma regions (south) and Singida and Shinyanga regions (west). Simanjiro District is one of the five districts of Manyara Region, others are: Babati, Kiteto, Hanang and Mbulu. According to the National Census (2002), the population of the district was around 141,676 (76,753 male and 64,923 female). Simanjiro District has twelve wards, which include Orkesumet, Naberera, Loibor-siret, Emboreet, Terrat, Oljoro-no 5, Shambarai, Mererani, Msitu wa Tembo, Ngorika, Ruvu- remit and Loiborsoit. The Mererani town is about 70km from city of Arusha, 20km from Kilimanjaro International Airport (KIA) and 120km from Orkesument, the Simanjiro District Headquarters. Nearly half of Simanjiro population resides in Mererani ward, which is a multi ethnic mining area with a range of people from most of Tanzania. The ward has five villages i.e. Songambele A, Songambele B, Zaire, Kazamoyo and Endiyamutu. Most of the Mererani ward is dry with significant deforestation and short periods of rain, generally there is a huge challenge of water shortage in the area. The area is estimated to harbour around 150,000 miners most of whom are constantly exposed to poor working conditions. But there are estimated 700 claim holders registered under Manyara Region Miners Association (MAREMA) whom about 400 are active miners, each with about 50 to 100 mineworkers. Hence total estimation of 21,000 to 42,000 mineworkers in total. TanzaniteOne is the only middle scale mining company in Mererani with about 400 employee (Mwaipopo-ako 1994, Bråtveit *et al.* 2003, Lange 2006, Kahuluda *et al.* 2010, Sosy 2013, Mpagama *et al.* 2015, Mayala *et al.* 2016).

The town has three health facilities located about 8 to 12 kilometers from the mines, one with GeneXpert service for TB diagnosis while the other two uses microscopy. There is neither nether X-ray nor spirometry services around the mining area.


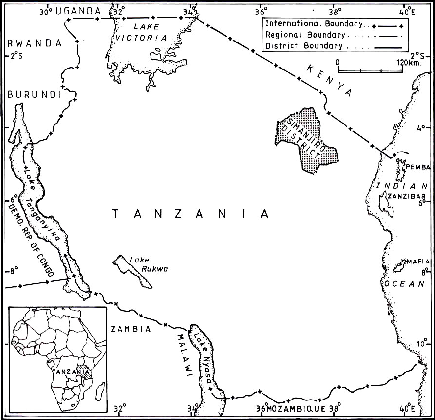


Figure 1: Location of Simanjiro District. Source: Yanda, P. Z. 2010 (62)

### Study population/area

The study will target tanzanite mining pits (work environment) in Mererani, Simanjaro District in which the mean concentrations of respirable crystalline silica and radon gas will be measured. It will also include the currently working SSM, those who goes down the mining pits and perform mining activities.

### Eligibility criteria

Inclusion criteria

- Tanzanite mining pits with currently running mining activities whose owners/managers have consented
- Consented tanzanite SSM aged at least 18 years
- Men aged at least 18 years engaged with other activities (e.g. teachers, health care providers, shopkeepers, etc.) other than mining from the general population surrounding the mining areas (for the comparison arm).

Exclusion criteria

- Partial exclusion for TB investigation using GeneXpert tests: SSM currently on TB treatment or completed TB treatment during the past 6 months
- Partial exclusion for lung function tests using spirometer: SSM found to have raised BP (systolic above 130mmHg and/or diastolic above 90mmHg) during initial assessment
- Men aged at least 18 years engaged with other activities (e.g. teachers, health care providers, shopkeepers, etc.) other than mining from the general population surrounding the mining areas but with history of working as a mine worker (for the comparison arm).

### Sample Size & Sampling

**Objective 1 &2:**

Among all active mining pits at the time of sample collection (estimated to be 70), 22 pits will be randomly selected from the list of all active mining pits. In each of these 22 pits, 3 air samples of at least 800litres (estimated working shift of 8 hours) each will be collected from the breathing zones of 3 randomly selected mineworkers from the list of all mine workers of the respective pit, hence a total of 2,400litres of air per pit. Radon gas concentrations will be measured both at the entrance and deep down of each of the 22 pits. Same approach and number of air samples (2,400litres) from 22 comparison sites in the general communities (from which also the comparison group of participants for investigation of silicosis, lung function and TB) will be taken. Under the guide of local government leaders in Mererani, a list of specific areas where there are pools of general community including schools, health facilities, markets, shops, etc. will be identified and listed. From the list, a random sample of 22 areas will be selected from which a list of men (as all mineworkers are men too) who are routine dwellers/workers will be obtained. From the list, a random sample of 3 individuals will be obtained from whom the air samples from their breathing zones will be collected. Teachers (not students), health care providers (not patients) and shopkeepers (not customers) will be the targeted.

**Objective 3,4 & 5**

From an estimated prevalence of silicosis of 1.6% among SSM in Tanzania (3 page 13), a basic sample of 206.4 was obtained. When adjusted by +10% for each of lung function assessment and TB investigation and addition +30% loss to follow up, the total sample comes to 325, approximated to 330 SSM at significance level α = 0.05. A size-proportion sample will be obtained from the randomly selected 22 mining pits. Same number of 330 participants (men) from the comparison group will be obtained from the general community. The comparison participants will be obtained from groups of general communities like health care providers in health facilities, teachers in primary and secondary schools, businessmen (shopkeepers) at the Mererani town. This sample will provide a reasonable estimate of the burden of silicosis; work related impaired lung function and TB among SSM in Mererani.

**Objective 6**

A total of 44 In-Depth Interview (IDI) will be conducted to 44 SSM obtained purposefully based on SSM working experience by selecting one most experienced and one least experienced from each of the 22 mining pits. Gatekeepers, likely the members of former TB patients’ non-governmental organizations working in the mining area will be used to get access to the appropriate interviewee.

### Study variables

The primary outcome variables will be the mean concentrations of respirable crystalline silica and radon gas among the SSM, and the miner’s perceptions and awareness regarding the health effects of exposure to the dust and gases.

Secondarily, the study will report on disease conditions related to mining occupation i.e. the burden of silicosis, TB and impaired lung function (both obstructive and restrictive lung diseases).

### Data tools & Study procedures

**Work Environment Assessment**

All SSM and their managers from the 22 selected pits and the individuals in the 22 selected comparison areas will be oriented on goal and objectives of the study, the dust sampling procedures with explanation that, and demonstration on how, some their fellow workers/friends need to be fitted with the personal dust sampler and also the measurements for radon gas that will be taken at their working places. This will be important so that all individuals in the same working area are aware of the study and should not get concerned/worried with some of them wearing the device while working. Each participants fitted with a personal dust sampler will be coded with respect to his specific mining pit or area of working for the comparison group. Personal samples of respirable dust will be collected during the working hours (routine shifts) using a calibrated DustTrak™ II Aerosol Monitor with Dorr-Oliver nylon cyclone fitted at the breathing zone of the participant. Air will be drawn using a portable battery operated personal pump fitted at the participants’ waist at a rate of 1.7l/min for the normal duration of the working shift, usually estimated to be 8hours (though may differ given a specific kind of work, especially for the comparison group). Throughout the air sample collection process and for all pumps, the same rate of 1.7l/min will be maintained. Samples for chemical analysis will be collected using a 37mm Polyvinyl Chloride (PVC) filter with pore size of 5μm. The filters will be put in the filter-cassettes, which will also be put in hard containers (to avoid damage) and transported to the laboratory for analysis. The personal dust sampler set will also record the prevailing temperature, pressure and humidity conditions. An expert in the area, likely an industrial hygienist will be hired/consulted to technically assist and guide throughout these processes.

The presence and concentrations of radon gas will be also be measured using AlphaGuard Monitor following the required standards and will be conducted by a hired technician from the Tanzania Atomic Energy Commission (TAEC). The measurements will be taken during working hours, from both the pits’ entrance and at the working sites down the pits.

**Diseases Screening and Investigation**

The selected and consented (after the consent has been read, counter-checked for understanding and signed) participants will be provided with a referral note and offered transport to KIDH at an estimated number of 25 to 30 participants per trip. At KIDH the participants will first pass through triaging in which those who appear to be in need urgent/immediate attention (if any) will be selected and offered the required service. Then the basic vital parameter, including BP, temperature, weight, height and oxygen saturation will be measured and documented. Then each participant will be taken through the pre-formed structured interview schedule that among others items, it will collect social-demographic data and the five TB screening questions as per National Tuberculosis and Leprosy Programme (NTLP).

All referred participants will be instructed on how to produce a quality sputum sample and will be provided with the chance to do so regardless of the TB screening status. Anyone whom thinks can produce sputum sample, will be provided with sputum container (Falcon tube) labelled with his specific identification number and directed to a specific pre-selected area for sputum production and collection. Once sputum sample has been collected, its specific laboratory form will be filled, packed and transported to the laboratory to be investigated using GeneXpert.

All the referred participants will perform a chest X-ray (Posterior Anterior view) using a digital DR GEM X-ray machine. Before the procedure, the participant will be shown and directed to the X-ray room with ensured privacy, where a trained radiographer will provide elaboration on the procedures for performing a chest X-ray including the need for the participant to remove his upper dressings (shirts, chains, etc.) and the need to perform and hold deep inhalation for a short time.

The participants will be undergoes assessment of their lung function using a Medical/Easy PC Spirometer. The spirometer will be calibrated on the morning of each clinic day before commencement of spirometry. The ambient temperature, pressure and humidity will be measured and documented into the Easy PC spirometer computer software.

All participants found to have silicosis, lung function impairment and/or TB, will be put under treatment and regular follow up as per the Ministry of Health, Community Development, Gender, Elderly and Children (MoHCDGEC) guidelines.

**Perception and Awareness**

The IDI will be done either in Mererani mines or at KIDH depending on easiness of execution. Pre-set IDI guides with probes will be used, following signing of the consent; the participant will be invited to a private and quite area prepared for the IDI. Rapport will be established and the participant will be introduced to the team and their specific roles during the interview including the moderator, notes taker and the timekeeper. The participant will be asked if has any question or concern of which may need clarification (if present it will be cleared). Then the audio recorder will be switched on and commence the interview following the IDI guide. After completion of the interview, the participant will be given a word of thanks for participating. Interview responses (notes) will be expanded and transcribed, themes of interest searched and coded. The summary notes and audio will be kept in a secure place.

**Pilot and Pre-testing of the data tools**

Pilot will be done to determine any unforeseen challenge(s) and rectify the gaps before engaging to the full study. This will involve going through all the study procedures for both the quantitative and qualitative parts, except shipping the dust samples to the laboratory for analysis (as even for the actual study, the dust samples will be packed and shipped to the accredited laboratory and will be processed under standardized procedures). One pit will be randomly selected from the list of all active pits (of which it won’t be included sampling list during the actual study) in which the at least two will be fitted with the personal dust sampler and collect samples of respirable crystalline silica and radon gas measurements from the same pit will be taken. The same will be done in the general community by randomly selecting one site from which at least two participants will be fitted with the personal dust sampler and collect air samples throughout a full working day.

The four participants (2 mine workers and 2 from general populations) will be consented for both the quantitative study (including the interview schedule) and the qualitative study (IDI). They will be offered transport to KIDH and undergo all the steps (and investigations) as per the actual study.

After completion of the pilot and pre-test of the tools, the investigator, the research assistants and key technical experts in the field of dust exposures and radians will jointly discuss and improve on the study procedures and the data collection tools so as to fit the actual study implementation.

### Data management & Analysis plan

Quantitative data will be summarized and cleaned using Microsoft Office Excel and then exported to Statistical Package for Social Sciences (SPSS) Statistics 25.0 (﻿IBM Corp., Armonk, NY, USA) for analysis. The mean concentrations of respirable crystalline silica and radon gas will be determined and compared between the two environments i.e. the mining pits and the general community by determining the Z-statistic, confidence interval and the associated p-value. The proportions of silicosis, TB and impaired lung functions will be determined in each of the mineworkers and the comparison group and the compared using Chi-squire test. The association between the exposures (respirable crystalline silica & radon gas) and occurrence of diseases (silicosis, impaired lung function & TB) in the two groups will be determined by logistic regression analysis. The chest X-rays will be interpreted independently by two radiologists with expertise and experience on interpretation of dust related lung diseases, including silicosis. In case of significant report discrepancies by the two radiologists, a third (more experienced) radiologist will be consulted to do the reading and the third report will be the final one. For the lung function assessment, the printed spirometry reports for each participant will be reviewed by two health care providers (likely registered nurses with a certificate on spirometry), and any discrepancies they will go back together and re-read the report for a final version.

The qualitative data will be analysed by determining the themes of interest, cut and joining related idea to synthesize and get meaning. Important quotes will be taken. Both manual approach and ENVIVO 12 (QSR International Pty Ltd, Victoria, Australia) computer software will be used to perform the analysis.

### Ethical consideration

Ethical clearance to conduct the study will be sought from the College Research and Ethical Review Committee of KCMUCo and the Institutional Review Board of KIDH. Permission to conduct the study will be sought from the Ministry of Minerals (Mererani Sub-Office), from the Regional Administrative Secretary (Manyara region), the District Executive Director (Simanjiro District) and Local Government Authorities in Mererani town. Also permission will be sought from the owners/managers for the specific mining companies.

Written informed consents, translated in Kiswahili language will be sought all individual participants, this will include consent for taking pictures (when needed). The participants will be identified and recorded using number and not their actual names for anonymity. The selected participants for undergoing investigations at KIDH will be provided with free transport from Mererani town to KIDH and back to Mererani town. A participant will have the right to withdrawal from the study at any time and this will not by any means affect the health services to be provided as may be needed.

### Plan of result dissemination

The research findings will be presented in various national and international conferences, mostly the Annual World Conferences on Lung Health (UNION Conferences) and KCMUCo academic fora. A minimum of three manuscripts will be written and submitted for publications in international journals, there will be under three main sub-topics:

- Work environment health risk factors: it will cover the findings on concentrations of respirable crystalline silica and radon gas, and also analysis of the chemical contents of the respirable dust.
- Work related diseases of the SSM: this will involve findings on the health state of the SSM with respect to prevalence of silicosis, impaired lung functions and tuberculosis.
- Awareness and perceptions: findings will entail the SSM awareness and perceptions on the health effects of being exposed to respirable crystalline silica.

Lastly, a PhD book will be written and submitted to KCMUCo.

### Study limitations

The following are the expected study limitations:

- No all miners will be able to undergo lung function tests, including miners who advanced impairing lung function or excessive cough as the test requires use of force to inhale and exhale. Also known patients with known pulmonary TB are not recommended to undergo the test while are still infectious.
- Some miners may decline to be investigated for TB due to the fear of being put out of work. Though efforts will be taken to educated and sensitize both the managers and the miners against this.
- Some managers may decline their workers to get out of work for the whole day so as to avoid decreased production. Efforts will be taken to educate the managers on the importance of study, including the free chance to evaluate the health of their workers and provide treatment to those found affected.

# PLAN OF ACTION

| Milestone | 2017/  2018 | 2019 | 2019 | 2019 | 2020 | 2020 |
| --- | --- | --- | --- | --- | --- | --- |
|  | Oct/Dec | Jan/Apr | May | Jun/Dec | Jan/Apr | Jan/June |
| Proposal development and approval |  |  |  |  |  |  |
| Training data collectors |  |  |  |  |  |  |
| Pilot & amendments |  |  |  |  |  |  |
| Data collection |  |  |  |  |  |  |
| Manuscripts writing & publications |  |  |  |  |  |  |
| Writing the PhD Book |  |  |  |  |  |  |
| Prepare further work for Post Doc |  |  |  |  |  |  |

# BUDGET

| **Ser No** | **Activities** | **Inputs required** | **Description** | **Meas. Unit** | **Unit cost (Tsh)** | **Number of units** | **Estimated cost (Tsh)** |
| --- | --- | --- | --- | --- | --- | --- | --- |
| 1.1 | To conduct assessment and documents about forms of exposure and types of respirable dust among tanzanite miners in Mererani, and lay down preventive and compensation recommendations for those affected | **Items & Supplies** |  |  |  |  |  |
|  |  | Pumps; hiring for 10 days | 10pumps*10 days*500,000Tsh | Each | 500,000 | 10 | 5,000,000 |
|  |  | PVC filter with cassettes | PVC filter | Pack (50) | 800,000 | 3 | 2,400,000 |
|  |  | Filter cassettes holder | 1packs (10)*1,326,000Tsh | Pack (10) | 1,326,000 | 1 | 1,326,000 |
|  |  | GS-1 Cyclone 10mm | 1packs (5)*1,808,625Tsh | Pack (5) | 1,808,625 | 1 | 1,808,625 |
|  |  | Tygon tubes | 5tubes*100,00Tsh | Each | 100,000 | 5 | 500,000 |
|  |  | AlphaGuard Monitor: Hiring for 10 days | 1machine*2,500,000Tsh | Each | 2,500,000 | 1 | 2,500,000 |
|  |  | Dry calibrator; hiring for 10 days | Calibrator | Each | 50,000 | 10 | 500,000 |
|  |  | **Collection of dust samples, gas measurements and conducting IDI** | | | | | - |
|  |  | Van hiring | 1van*400,000Tsh*10 days | Days | 400,000 | 10 | 4,000,000 |
|  |  | Transportation refund for participants | 660persons*3,000Tsh | Person | 3,000 | 660 | 1,980,000 |
|  |  | Supervisor air ticket - return | 1person*550,000Tsh | Person | 550,000 | 1 | 550,000 |
|  |  | Perdiems; Supervisor (MUHAS) | 1person*120,000Tsh*3days | Days | 120,000 | 3 | 360,000 |
|  |  | Perdiems; KIDH staff | 1persons*120,000Tsh*15days | Days | 120,000 | 10 | 1,200,000 |
|  |  | Perdiems; Driver | 1person*100,000Tsh*15days | Days | 100,000 | 10 | 1,000,000 |
|  |  | Perdiems; Data/sample collector | 1persons*100,000Tsh*15days | Days | 100,000 | 10 | 1,000,000 |
|  |  | Perdiems - DTLC - Simanjiro District | 1person*100,000Tsh*3days | Days | 100,000 | 5 | 500,000 |
|  |  | Laboratory dust analysis fees | 150samples* | Each | 50,000 | 150 | 7,500,000 |
|  |  | Perdimes; Technician from TAEC | 1person*120,000Tsh*10 days | Days | 120,000 | 10 | 1,200,000 |
|  |  | Publications | 3papers*2,000,000Tsh | Each | 2,000,000 | 3 | 6,000,000 |
|  | **Total** |  |  |  |  |  | **39,324,625** |

# REFERENCES

Alif, S.M., Dharmage, S.C., Bowatte, G., Karahalios, A., Benke, G., Dennekamp, M., Mehta, A.J., Miedinger, D., Künzli, N., Probst-Hensch, N., and Matheson, M.C., 2016. Occupational exposure and risk of chronic obstructive pulmonary disease: a systematic review and meta-analysis. *Expert Review of Respiratory Medicine*, 10 (8), 861–872.

Ameille, J., Letourneux, M., Paris, C., Brochard, P., Stoufflet, A., Schorle, E., Gislard, A., Laurent, F., Conso, F., and Pairon, J.C., 2010. Does asbestos exposure cause airway obstruction, in the absence of confirmed asbestosis? *American Journal of Respiratory and Critical Care Medicine*, 182 (4), 526–530.

Ayaaba, E., Li, Y., Yuan, J., and Ni, C., 2017. Occupational respiratory diseases of miners from two gold mines in Ghana. *International Journal of Environmental Research and Public Health*, 14 (3).

Blanc, P.D. and Torén, K., 2016. COPD and occupation: Resetting the agenda. *Occupational and Environmental Medicine*, 73 (6), 357–358.

Bolund, A.C.S., Miller, M.R., Sigsgaard, T., and Schlünssen, V., 2017. The effect of organic dust exposure on long-term change in lung function: A systematic review and meta-analysis. *Occupational and Environmental Medicine*, 74 (7), 531–542.

Boniface, R., Museru, L., Munthali, V., and Lett, R., 2013. Occupational injuries and fatalities in a tanzanite mine: Need to improve workers safety in Tanzania. *The Pan African medical journal*, 16, 120.

Borup, H., Kirkeskov, L., Hanskov, D.J.A., and Brauer, C., 2017. Systematic review: chronic obstructive pulmonary disease and construction workers. *Occupational Medicine*, 67, 199–204.

Bråtveit, M., Moen, B.E., Mashalla, Y.J.S., and Maalim, H., 2003. Dust exposure during small-scale mining in Tanzania: A pilot study. *Annals of Occupational Hygiene*, 47 (3), 235–240.

Brueske, I., Thiering, E., Heinrich, J., Huster, K.M., and Nowak, D., 2014. Respirable quartz dust exposure and airway obstruction: a systematic review and meta-analysis. *Occupational and Environmental Medicine*, 71 (8), 583–589.

Brüske, I., Thiering, E., Heinrich, J., Huster, K., and Nowak, D., 2013. Biopersistent granular dust and chronic obstructive pulmonary disease: A systematic review and meta-analysis. *PLoS ONE*, 8 (11), 1–11.

Chen, M., 2012. Laryngeal Cancer and Silica Dust Exposure : A Systematic Review and Meta-Analysis. *American Journal of Industrial Medicine*, 676, 669–676.

Chen, W., Liu, Y., Wang, H., Hnizdo, E., Sun, Y., Su, L., Zhang, X., Weng, S., Bochmann, F., Hearl, F.J., Chen, J., and Wu, T., 2012. Long-term exposure to silica dust and risk of total and cause-specific mortality in Chinese workers: A cohort study. *PLoS Medicine*, 9 (4).

Chen, W., Zhuang, Z., Attfield, M.D., Chen, B.T., Gao, P., Harrison, J.C., Fu, C., Chen, J.Q., and Wallace, W.E., 2001. Exposure to silica and silicosis among tin miners in China: Exposure-response analyses and risk assessment. *Occupational and Environmental Medicine*, 58 (1), 31–37.

Collins, J.F., Salmon, A.G., Brown, J.P., Marty, M.A., and Alexeeff, G. V., 2005. Development of a chronic inhalation reference level for respirable crystalline silica. *Regulatory Toxicology and Pharmacology*, 43 (3), 292–300.

Cumberbatch, M.G.K., Cox, A., Teare, D., and Catto, J.W.F., 2015. Contemporary occupational carcinogen exposure and bladder cancer. *JAMA Oncology*, 1 (9), 1282–1290.

Dahmann, D., Taeger, D., Kappler, M., Büchte, S., Morfeld, P., Brüning, T., and Pesch, B., 2008. Assessment of exposure in epidemiological studies: The example of silica dust. *Journal of Exposure Science and Environmental Epidemiology*, 18 (5), 452–461.

Doust, E., Ayres, J.G., Devereux, G., Dick, F., Crawford, J.O., Cowie, H., and Dixon, K., 2014. Is pesticide exposure a cause of obstructive airways disease? *European Respiratory Review*, 23 (132), 180–192.

Eftimie, A., Heller, K., Strongman, J., Hinton, J., Lahiri-Dutt, K., and Mutemeri, N., 2012. Gender Dimensions of Artisanal and Small-Scale Mining. A Rapid Assessment Toolkit Adriana. The World Bank.

Fisher, E., 2007. Occupying the margins: Labour integration and social exclusion in artisanal mining in Tanzania. *Development and Change*, 38 (4), 735–760.

Fishwick, D., Sen, D., Barber, C., Bradshaw, L., Robinson, E., Sumner, J., Hoyle, J., Mayatt, V., Stenton, C., Warburton, C., and Burge, S., 2015. Occupational chronic obstructive pulmonary disease: A standard of care. *Occupational Medicine*, 65 (4), 270–282.

Flynn, M.R. and Susi, P., 2003. Engineering controls for selected silica and dust exposures in the construction industry - A review. *Applied Occupational and Environmental Hygiene*, 18 (4), 268–277.

Fu, N.F., Luo, C.H., Wu, J.C., Zheng, Y.Y., Gan, Y.J., Ling, J.A., Liang, H.Q., Liang, D.Y., Xie, J., Chen, X.Q., Li, X.J., Pan, R.H., Chen, Z.X., and Jiang, S.J., 2013. Clearance of free silica in rat lungs by spraying with Chinese herbal kombucha. *Evidence-based Complementary and Alternative Medicine*, 2013.

Garshick, E., Schenker, M.B., and Dosman, J.A., 1996. Occupationally induced airways obstruction. *Medical Clinics of North America*, 80 (4), 851–878.

Gottesfeld, P., Andrew, D., and Dalhoff, J., 2015. Silica Exposures in Artisanal Small-Scale Gold Mining in Tanzania and Implications for Tuberculosis Prevention. *Journal of Occupational and Environmental Hygiene*, 12 (9), 647–653.

Hutyrová, B., Smolková, P., Nakládalová, M., Tichý, T., and Kolek, V., 2015. Case of accelerated silicosis in a sandblaster. *Industrial Health*, 53 (2), 178–183.

IOM, n.d. TB / HIV Vulnerabilities in the Mining Sector in Tanzania : Report of the National Consultation on TB in the Mining Sector in Tanzania and Rapid Baseline Assessment on TB in the Mining Industry in Mererani Town, Geita Region and Kishapu District, 1–54.

Kabir, E., Islam, A., and Taufikuzzaman, M., 2018. An investigation into respiratory health problems of workers at stone crushing industries in Bangladesh. *Journal of Health Research*, 32 (2), 172–178.

Kahuluda, E.M., Makundi, I.N., Directorate, R.C., and Commission, E., 2010. Determination of Radon Gas and Respirable Ore Dust Concentrations in the Underground Merelani Tanzanite, (Mudd 2008).

Kraïm-Leleu, M., Lesage, F.X., Drame, M., Lebargy, F., and Deschamps, F., 2016. Occupational risk factors for COPD: A case-control study. *PLoS ONE*, 11 (8), 1–11.

Lange, S., 2006. Benefit streams from mining in Tanzania: Case Studies from Geita and Mererani. CMI.

Maciejewska, A., 2015. Health effects of occupational exposure to crystalline silica in the light of current research results. *Medycyna Pracy*, 65 (6), 799–818.

Madl, A.K., Donovan, E.P., Gaffney, S.H., McKinley, M.A., Moody, E.C., Henshaw, J.L., and Paustenbach, D.J., 2008. State-of-the-Science Review of the Occupational Health Hazards of Crystalline Silica in Abrasive Blasting Operations and Related Requirements for Respiratory Protection. *Journal of Toxicology and Environmental Health - Part B: Critical Reviews*, 11 (7), 548–608.

Malisa, E.P. and Kinabo, C.P., 2005. Environmental risks for gemstone miners with reference to Merelani Tanzanite mining area, Northeastern Tanzania. *Tanzania Journal of Science*, 31 (01), 1–12.

de Matteis, S., Heederik, D., Burdorf, A., Colosio, C., Cullinan, P., Henneberger, P.K., Olsson, A., Raynal, A., Rooijackers, J., Santonen, T., Sastre, J., Schlünssen, V., Tongeren, M. Van, and Sigsgaard, T., 2017. Current and new challenges in occupational lung diseases. *European Respiratory Review*, 26 (146), 1–15.

De Matteis, S., Jarvis, D., Hutchings, S., Darnton, A., Fishwick, D., Sadhra, S., Rushton, L., and Cullinan, P., 2016. Occupations associated with COPD risk in the large population-based UK Biobank cohort study. *Occupational and Environmental Medicine*, 73 (6), 378–384.

Mayala, L.P., Veiga, M.M., and Khorzoughi, M.B., 2016. Assessment of mine ventilation systems and air pollution impacts on artisanal tanzanite miners at Merelani, Tanzania. *Journal of Cleaner Production*, 116, 118–124.

Mdee, O.J., 2015. Potential of artisanal and small-scale gold mines for economic development in Tanzania: A review. *Journal of Geology and Mining Research*, 7 (2), 46–56.

Merget, R., Bauer, T., Küpper, H., Philippou, S., Bauer, H., Breitstadt, R., and Bruening, T., 2002. Health hazards due to the inhalation of amorphous silica. *Archives of Toxicology*, 75 (11), 625–634.

Mohammadyan, M., Rokni, M., and Yosefinejad, R., 2013. Occupational exposure to respirable crystalline silica in the Iranian mazandaran province industry workers. *Arhiv za Higijenu Rada i Toksikologiju*, 64 (1), 139–143.

Möhner, M., Pohrt, A., and Gellissen, J., 2017. Occupational exposure to respirable crystalline silica and chronic non-malignant renal disease: systematic review and meta-analysis. *International Archives of Occupational and Environmental Health*, 90 (7), 555–574.

Mpagama, S.G., Lekule, I.A., Mbuya, A.W., Kisonga, R.M., and Heysell, S.K., 2015. The influence of mining and human immunodeficiency virus infection among patients admitted for retreatment of tuberculosis in Northern Tanzania. *American Journal of Tropical Medicine and Hygiene*, 93 (2), 212–215.

Mwaipopo-ako, R., 1994. The Impact of Technology on Poverty Alleviation: The Case of Artisanal Mining in Tanzania By State Mining Corporation , Dar es Salaam.

Nakládalová, M., Štěpánek, L., Kolek, V., Žurková, M., and Tichý, T., 2018. A case of accelerated silicosis. *Occupational Medicine*, 68 (7), 482–484.

Nelson, G., 2012. Living in the Shadow of a Dust Cloud: Occupational respiratory diseases in the South African mining industry, 1975 to 2009. University of the Witwatersrand.

Ngosa, K. and Naidoo, R.N., 2016. The risk of pulmonary tuberculosis in underground copper miners in Zambia exposed to respirable silica: A cross-sectional study. *BMC Public Health*, 16 (1), 1–7.

Osman, S., Ziegler, C., Gibson, R., Mahmood, R., and Moraros, J., 2017. Original Article The Association between Risk Factors and Chronic Obstructive Pulmonary Disease in Canada: A Cross‑sectional Study Using the 2014 Canadian Community Health Survey. *International Journal of Preventive Medicine*, 8 (86).

Oxman, A.D., Muir, D.C.F., Shannon, H.S., Stock, S.R., Hnizdo, E., and Lange, H.J., 1993. Occupational Dust Exposure and Chronic Obstructive Pulmonary Disease: A Systematic Overview of the Evidence. *American Review of Respiratory Disease*, 148 (1), 38–48.

Pius, Z.Y., a, and Christopher, W., 2010. Livelihoods diversifications and implications on food security and poverty levels in the Maasai plains: The case of Simanjiro district, Northern Tanzania. *African Journal of Environmental Science and Technology*, 4 (3), 154–166.

Quinlan, P.J., Earnest, G., Eisner, M.D., Yelin, E.H., Katz, P.P., Balmes, J.R., and Blanc, P.D., 2009. Performance of self-reported occupational exposure compared to a job-exposure matrix approach in asthma and chronic rhinitis. *Occupational and Environmental Medicine*, 66 (3), 154–160.

Reiprich, G., van Zyl, A., Govender, V., Kamadu, A., Lloyd, A., and Reddy, C., n.d. *Service Package F: Prevention of TB in the mines Consolidated Report: Review of existing legislation and regulations for mine health and safety in the ten countries*. Health Focus.

Sadhra, S., Kurmi, O.P., Sadhra, S.S., Lam, K.B.H., and Ayres, J.G., 2017. Occupational COPD and job exposure matrices: A systematic review and meta-analysis. *International Journal of COPD*, 12, 725–734.

Sen, S., Mitra, R., Mukherjee, S., K. Das, P., and Moitra, S., 2016. Silicosis in Current Scenario: A Review of Literature. *Current Respiratory Medicine Reviews*, 12 (1), 56–64.

Sharma, N., Kundu, D., and Das, A., 2016. WHO | Silicosis and silicotuberculosis in India. *Who*, (February), 777–778.

Skogstad, M., Johannessen, H.A., Tynes, T., Mehlum, I.S., Nordby, K.C., and Lie, A., 2016. Systematic review of the cardiovascular effects of occupational noise. *Occupational Medicine*, 66 (6), 500–500.

Sosy, J.J., 2013. Socio-Economic Constraints among Local Artisanal Miners in Simanjiro District, Tanzania. Sokoine University of Agriculture. Morogoro, Tanzania.

Steenland, K., Mannetje, A., Boffetta, P., Stayner, L., Attfield, M., Chen, J., Dosemeci, M., DeKlerk, N., Hnizdo, E., Koskela, R., and Checkoway, H., 2001. Pooled exposure-response analyses and risk assessment for lung cancer in 10 cohorts of silica-exposed workers: An IARC multicentre study. *Cancer Causes and Control*, 12 (9), 773–784.

Swanepoel, A.J., Rees, D., Renton, K., Swanepoel, C., Kromhout, H., and Gardiner, K., 2010. Quartz exposure in agriculture: Literature review and South African survey. *Annals of Occupational Hygiene*, 54 (3), 281–292.

TIMS, 2017. Report of the Dissemination Workshop for the Evidence Generating Studies Conducted Under the TIMS Grant.

UN Women, 2016. Mapping Study on Gender and Extractive Industries in Mainland Tanzania, (November).

Vinnikov, D., 2016. Drillers and mill operator in an open-pit gold mine are at risk for impaired lung function. *Journal of Occupational Medicine and Toxicology*, 11 (1), 1–6.

van der Wal, S. and Haan, E. de, 2010. Rough Cut: Sustainability Issues in the Coloured Gemstone Industry.

# ANNEXES

## Data Collection Tools

### Interview Schedule – English Version

**SILICA AND RADON EXPOSURES AND ITS ASSOCIATED EFFECTS ON THE RESPIRATORY SYSTEM AMONG SMALL SCALE MINERS IN MERERANI**

**Interview Schedule – English Version**

**(*Adapted from: Medical Research Council (UK) Respiratory Questionnaire, 1986*)**

**Preamble:** I am going to ask some general questions but also about any breathing or respiratory symptoms.

|  | Interviewer’s ID:  Participant’s ID:  Date of interview: (dd/mm/yyyy) | ____________________  ____________________  ____________________ |  |
| --- | --- | --- | --- |
| **Ser No** | **Question** | **Response** | **Code** |
|  | **LOCATION** |  |  |
| 1 | District |  | L1 |
| 2 | Ward |  | L2 |
| 3 | Village Name |  | L3 |
| 4 | Village ID | \|  \|  \|  \|  \| \| --- \| --- \| --- \| --- \| | L4 |
| 5 | Pit Name |  | L5 |
| 6 | Pit ID | \|  \|  \|  \| \| --- \| --- \| --- \| | L6 |
| 7 | GPS | \|  \|  \|  \|  \|  \|  \|  \|  \| \| --- \| --- \| --- \| --- \| --- \| --- \| --- \| --- \| | L7 |
|  | **DEMOGRAPHY** |  |  |
| 8 | Sex (*record as observed*) | Male: 1  Female: 2 | D1 |
| 9 | How old are you? | Years: _____(*years*) | D2 |
| 10 | What is the highest level of education you completed? | No formal education: 1  Less than primary school: 2  Completed primary school: 3  Completed secondary school: 4  Above secondary school: 5  Refuse to respond: 77 | D3 |
| 11 | What is your region/district of birth? | Region: ___________________  District: ___________________ | D4 |
| 12 | During the past one-year, how many times have you visited your relatives at your region/district of birth? | ________________ | D5 |
| 13 | What is your marital status | Never married: 1  Currently married: 2  Separated: 3  Divorced: 4  Widowed: 5  Cohabiting: 6  Refused to respond: 77 | D6 |
| 14 | For how long have you work as a tanzanite mineworker in Mererani? | ___________________ (years) | D7 |
| 15 | Have you worked as a mineworker in any other areas other than Mererani? | Yes: 1  No: 2 | D8 |
| 16 | ***If yes to Question 15***; for how long have you work as mineworker in other areas other than Mererani? | ___________________ (years) | D9 |
| 17 | What is your average income per month? | ____________________ (Tsh) | D10 |
|  | **SYMPTOMATIC SCREENING OF TUBERCULOSIS** |  |  |
| 18 | Have you had cough for the past two weeks? | Yes: 1  No: 2 | SS1 |
| 19 | Have you had excessive night sweating for the past two weeks? | Yes: 1  No: 2 | SS2 |
| 20 | Have you had significant weight loss (at least 3 kg) in the last 3 months? | Yes: 1  No: 2 | SS3 |
| 21 | Have you noted blood in sputum (or cough up blood)? | Yes: 1  No: 2 | SS4 |
| 22 | Have you had chronic fever for the past two weeks? | Yes: 1  No: 2 | SS5 |
|  | *If the subject is disabled from walking from any condition other than heart and lung disease, please begin questionnaire at* ***Question 27*** |  |  |
|  | **BREATHLESSNESS AND WHEEZING** |  |  |
|  | **During the last month:** |  |  |
| 23 | Are you troubled by shortness of breath when hurrying on level ground or walking up a slight hill? | Yes: 1  No: 2 | B1 |
| 24 | Do you get short of breath walking with other people of your age on level ground? | Yes: 1  No: 2 | B2 |
| 25 | Do you have to stop for breath when walking at your own pace on level ground? | Yes: 1  No: 2 | B3 |
| 26 | If you run, or climb stairs fast do you ever: |  |  |
|  | a) Cough? | Yes: 1  No: 2 | B4a |
|  | b) Wheeze? | Yes: 1  No: 2 | B4b |
|  | c) Get tight in the chest? | Yes: 1  No: 2 | B4c |
| 27 | Is your sleep ever broken: |  |  |
|  | a) By wheeze? | Yes: 1  No: 2 | B5a |
|  | b) Difficulty in breathing? | Yes: 1  No: 2 | B5b |
| 28 | Do you ever wake up in the morning |  |  |
|  | a) With wheeze? | Yes: 1  No: 2 | B6a |
|  | b) With difficulty in breathing? | Yes: 1  No: 2 | B6b |
| 29 | Do you ever wheeze? |  |  |
|  | a) If you are in a smoky room? | Yes: 1  No: 2 | B7a |
|  | b) If you are in a very dusty place? | Yes: 1  No: 2 | B7b |
| 30 | ***If you have answered yes to any of Questions 27-29, are your symptoms better:*** |  |  |
|  | a) At weekends? | Yes: 1  No: 2 | B8a |
|  | b) Extended times when away from work? | Yes: 1  No: 2 | B8b |
| 31 | ***If Yes to Question 30***, please record details of any occupational exposure to other respiratory hazards, e.g., isocyanates, wood dust, or formaldehyde |  | B9 |
|  | **COUGH** |  |  |
| 32 | Do you usually cough first thing in the morning in winter? | Yes: 1  No: 2 | C1 |
| 33 | Do you usually cough during the day – or at night – in the winter? | Yes: 1  No: 2 | C2 |
|  | ***If you have answered yes to Questions 32 or 33***, do you cough like this on most days for as much as three months each year? | Yes: 1  No: 2 | C3 |
|  | **PHLEGM** |  |  |
| 34 | Do you usually bring up phlegm from your chest first thing in the morning in winter? | Yes: 1  No: 2 | P1 |
| 35 | Do you usually bring up any phlegm from your chest during the day – or at night – in winter? | Yes: 1  No: 2 | P2 |
| 36 | ***If you have answered yes to Questions 34 or 35***, do you bring up phlegm like this on most days for as much as three months each year? | Yes: 1  No: 2 | P3 |
|  | **PERIODS OF COUGH AND PHLEGM** |  |  |
| 37 | In the past three years, have you had a period of (increased) cough and phlegm lasting for three weeks or more? | Yes: 1  No: 2 | CP1 |
| 38 | ***If you have answered yes to Question 37***, have you had more than one such episode? | Yes: 1  No: 2 | CP2 |
|  | **CHEST ILLNESSES** |  |  |
| 39 | During the past three years, have you had any chest illness that has kept you from your usual activities for as much as a week? | Yes: 1  No: 2 | CH1 |
| 40 | ***If you have answered yes to Question 39***, Did you bring up more phlegm than usual in any of these illnesses? | Yes: 1  No: 2 | CH2 |
| 41 | ***If you have answered yes to Question 40***, Have you had more than one illness like this in the past three years? | Yes: 1  No: 2 | CH3 |
|  | **PAST ILLNESSES** |  |  |
| 42 | Have you ever had, or been told (at hospital) that you have had: |  |  |
|  | a) An injury, or operation affecting your chest? | Yes: 1  No: 2 | PA1a |
|  | b) Heart trouble? | Yes: 1  No: 2 | PA1b |
|  | c) Bronchitis? | Yes: 1  No: 2 | PA1c |
|  | d) Pneumonia? | Yes: 1  No: 2 | PA1d |
|  | e) Pleurisy? | Yes: 1  No: 2 | PA1e |
|  | f) Asthma? | Yes: 1  No: 2 | PA1f |
|  | g) Other chest trouble | Yes: 1  No: 2 | PA1g |
|  | h) Hay fever | Yes: 1  No: 2 | PA1h |
|  | i) Tuberculosis | Yes: 1  No: 2 | PA1i |
|  | j) Silicosis | Yes: 1  No: 2 | PA1j |
|  | **TOBACCO SMOKING** |  |  |
| 43 | Do you smoke? | Yes: 1  No: 2 | TS1 |
| 44 | ***If you answered No to Question 43***, have you ever smoked as much as one cigarette a day for as long as one year? | Yes: 1  No: 2 | TS2 |
|  | ***If you answered No to Question 43 or 44, omit remaining questions.*** |  |  |
| 45 | How old were you when you started smoking regularly? | ______(*years*) | TS3 |
| 46 | Do (did) you smoke manufactured cigarettes? | Yes: 1  No: 2 | TS4 |
| 47 | ***If you answered yes to Question 46:*** |  |  |
|  | a) How many do you (did) usually smoke per day? | ______ (*actual number*) | TS4a |
|  | b) On weekdays | ______ (*actual number*) | TS4b |
|  | c) At weekends | ______ (*actual number*) | TS4c |
| 48 | Do you smoke any other forms of tobacco? | Yes: 1  No: 2 | TS5 |
| 49 | ***If Yes to Question 48: Record details under additional notes.*** | *Additional notes*:  ____________________________  __________________________  __________________________  __________________________  __________________________  __________________________ | TS6 |
| 50 | When did you give up smoking altogether? | ______________(*month/year*) | TS7 |

***Additional Notes*** *This questionnaire is based on the MRC (UK) Respiratory Questionnaire 1986, which has been extensively validated. This questionnaire will be completed by a worker with the assistance of a native speaker. Additional questions have been added to cover clinical aspects of bronchial hyper responsiveness validated by the Department of Occupational and Environmental Medicine, National Lung Institute. The British Occupational Health Research Foundation (BOHRF) concluded that in the clinical setting questionnaires that identify symptoms of wheeze and/or shortness of breath which improve on days away from work or on holidays have a high sensitivity, but relatively low specificity for occupational asthma.*

### Interview Schedule – Swahili Version

**UVUTAJI WA VUMBI LA SILICA NA GESI YA RADON NA MADHARA YAKE KATIKA MFUMO WA UPUMUAJI KWA WACHIMBAJI WADOGO WA MADINI YA TANZANITE, MERERANI**

**Ratiba ya Mahojiano – Swahili Version**

**(*Imetoholewa kutoka: Medical Research Council (UK) Respiratory Questionnaire, 1986*)**

Utangulizi: Nitakuuliza maswali ya jumla na pia maswali dalili zinazohusu upumuaji na mfumo wa upumuaji.

|  | Utambulisho wa Mhoji:  Utambulisho wa Muhojiwa:  Tarehe ya Mahojiano: (ss/mm/mmmm) | ____________________  ____________________  ____________________ |  |
| --- | --- | --- | --- |
| **Nm** | **Swali** | **Jibu** | **Alama Siri** |
|  | **ENEO** |  |  |
| 1 | Wilaya |  | L1 |
| 2 | Kata |  | L2 |
| 3 | Kijiji |  | L3 |
| 4 | Utambulisho wa Kijiji | \|  \|  \|  \|  \| \| --- \| --- \| --- \| --- \| | L4 |
| 5 | Jina la Mgodi |  | L5 |
| 6 | Utambulisho wa Mgodi | \|  \|  \|  \| \| --- \| --- \| --- \| | L6 |
| 7 | GPS | \|  \|  \|  \|  \|  \|  \|  \|  \| \| --- \| --- \| --- \| --- \| --- \| --- \| --- \| --- \| | L7 |
|  | **DEMOGRAFIA** |  |  |
| 8 | Jinsia (*kwa muonekano*) | Mwanaume: 1  Mwanamke: 2 | D1 |
| 9 | Una umri wa miaka mingapi? | _____(*miaka*) | D2 |
| 10 | Ni kiwango kipi cha juu cha elimu ulichowahi kufikia? | Hajahudhuria shule rasmi: 1  Chini ya elimu ya msingi: 2  Amekamilisha elimu ya msingi: 3  Amekamilisha elimu ya sekondari: 4  Zaidi ya elimu ya sekondari: 5  Amekataa kujibu: 77 | D3 |
| 11 | Umezaliwa mkoa/wilaya gani? | Mkoa: ___________________  Wilaya: __________________ | D4 |
| 12 | Katika mwaka mmoja uliopita, ni mara ngapi ummenda kuwaona ndugu zako katika mkoa/wilaya uliozaliwa? | ________________ | D5 |
| 13 | Upo katika hali gani ya ndoa/mahusiano | Hajawahi kuwa na ndoa: 1  Yupo kwenye ndoa: 2  Wameachana bila talaka: 3  Wametalakiana: 4  Mjane/Mgane: 5  Yupo kwenye mahusiano: 6  Amekataa kujibu: 77 | D6 |
| 14 | Umefanya kazi ya uchimbaji madini ya tanzanite kwa muda gani? | ___________________ (*miaka*) | D7 |
| 15 | Umewahi kufanya kazi ya kuchimba madini eneo jingine/maeneo mengine zaidi ya Mererani? | Ndio: 1  Hapana: 2 | D8 |
| 16 | ***Kama jibu ni ndio kwa swali nm 15;*** umekuwa mchimbaji kwa muda gani katika eneo/maeneo mengine zaidi ya Mererani? | ___________________ (*miaka*) | D9 |
| 17 | Kwa wastani, kipato chako kwa mwezi ni kiasi gani? | ____________________ (Tsh) | D10 |
|  | **UCHUNGUZI WA DALILI ZA KIFUA KIKUU** |  |  |
| 18 | Je, unakohoa kwa wiki mbili au zaidi? | Ndio: 1  Hapana: 2 | SS1 |
| 19 | Je, unatokwa na jasho jingi kwa wiki mbili au zaidi? | Ndio: 1  Hapana: 2 | SS2 |
| 20 | Je, umepoteza uzito katika mwezi mmoja uliopita? | Ndio: 1  Hapana: 2 | SS3 |
| 21 | Je, umeona damu katika makohozi au kukohoa damu? | Ndio: 1  Hapana: 2 | SS4 |
| 22 | Je, unapatwa na homa za mara kwa mara katika wiki mbili zilizopita? | Ndio: 1  Hapana: 2 | SS5 |
|  | *Endapo mhojiwa anapata changamoto katika kutembea kutokana na tatizo lolote/ugonjwa wowote zaidi ya tatizo/ugonjwa wa moyo and mapafu, anza dodoso katika swali la 27* |  |  |
|  | **PUMZI KUBANA NA SAUTI YA FILIMBI** |  |  |
|  | **Katika mwezi mmoja uliopita:** |  |  |
| 23 | Je, unabanwa na pumzi ukitembea kwa haraka katika eneo tambarare au ukipanda sehemu yenye muinuko? | Ndio: 1  Hapana: 2 | B1 |
| 24 | Je, unabanwa na pumzi ukiwa unatembea na watu wengine wa umri kama wako katika eneo tambarare? | Ndio: 1  Hapana: 2 | B2 |
| 25 | Je, unalazimika kusimama kupumua vizuri ukiwa unatembea kwa mwendo wa kawaida katika sehemu tambarare? | Ndio: 1  Hapana: 2 | B3 |
| 26 | Je, ukikimbia au kupanda ngazi kwa haraka, unapatwa na: |  |  |
|  | a) Kikohozi? | Ndio: 1  Hapana: 2 | B4a |
|  | b) Kupumua kwa sauti ya filimbi? | Ndio: 1  Hapana: 2 | B4b |
|  | c) Kifua kinabana? | Ndio: 1  Hapana: 2 | B4c |
| 27 | Je, unakatishwa usingizi na: |  |  |
|  | a) Kutoa sauti ya filimbi ukipumua? | Ndio: 1  Hapana: 2 | B5a |
|  | b) Kubanwa na pumzi? | Ndio: 1  Hapana: 2 | B5b |
| 28 | Je, huwa unaamka asubuhi ukiwa na: |  |  |
|  | a) Kupumua kwa sauti ya filimbi? | Ndio: 1  Hapana: 2 | B6a |
|  | b) Kubanwa na pumzi? | Ndio: 1  Hapana: 2 | B6b |
| 29 | Je, ulishapatwa na hali ya kupumua kwa sauti ya filimbi: |  |  |
|  | a) Kama ukiwa kwenye chumba chenye moshi? | Ndio: 1  Hapana: 2 | B7a |
|  | b) Kama ukiwa katika eneno lenye vumbi jingi? | Ndio: 1  Hapana: 2 | B7b |
| 30 | ***Kama umejibu NDIO kwa swali lolote kati ya swali la 27-29, Je, unapata unafuu:*** |  |  |
|  | a) Wakati wa mwisho wa wiki? | Ndio: 1  Hapana: 2 | B8a |
|  | b) Muda baada ya kumaliza kazi zako? | Ndio: 1  Hapana: 2 | B8b |
| 31 | ***Kama jibu ni NDIO katika swali la 30***, tafadhali elezea athari zozote za mfumo wa upumuaji zinazotokana na vichochezi katika mazingira ya kazi, mfano ‘isocyanates’, vumbi la mbao au ‘formaldehyde’ |  | B9 |
|  | **KIKOHOZI** |  |  |
| 32 | Je, wakati wa msimu wa baridi uwa unakohoa mapema kabisa asubuhi | Ndio: 1  Hapana: 2 | C1 |
| 33 | Je, wakati wa msimu wa baridi, ni kawaida kuwa unakohoa wakati wa mchana au wakati wa usiku? | Ndio: 1  Hapana: 2 | C2 |
|  | ***Kama amejibu NDIO kwa swali la 32 au 33,*** Je, unakohoa kwa namna hii kwa siku nyingi, kwa kipindi cha kufikia miezi mitatu kwa kila mwaka? | Ndio: 1  Hapana: 2 | C3 |
|  | **MAKOHOZI** |  |  |
| 34 | Je, katika msimu wa baridi ni mara nyingi unatoa makohozi kutoka ndani ya kifua mapema asubuhi? | Ndio: 1  Hapana: 2 | P1 |
| 35 | Je, katika msimu wa baridi huwa ni mara nyingi unatoa makohozi kutoka ndani ya kifua? | Ndio: 1  Hapana: 2 | P2 |
| 36 | ***Kama umejibu NDIO kwa swali la 34 au 35,*** Je, huwa unatoa makohozi namna hio kwa siku nyingi katika muda unaofikia miezi mitatu kila mwaka? | Ndio: 1  Hapana: 2 | P3 |
|  | **VIPINDI VYA KUKOHOA NA MAKOHOZI** |  |  |
| 37 | Katika muda wa miaka mitatu iliopita, umeshakuwa na vipindi vya (kuongezeka) kwa kikohozi na kutoa makohozi, vinavyokuwepo kwa muda wa wiki tatu au zaidi? | Ndio: 1  Hapana: 2 | CP1 |
| 38 | ***Kama umejibu NDIO kwa swali la 37,*** Je, umeshapatwa na vipindi zaidi ya kimoja vya hali hio? | Ndio: 1  Hapana: 2 | CP2 |
|  | **MAGONJWA YA KIFUA** |  |  |
| 39 | Katika miaka mitatu iliopita, umeshapatwa na ugonjwa wa kifua uliosababisha ushindwe kufanya kazi zako kwa muda unaofikia wiki moja? | Ndio: 1  Hapana: 2 | CH1 |
| 40 | ***Kama umejibu NDIO katika swali la 39,*** Je, ulitoa makohozi mengi zaidi ha kawaida wakati ka ugonjwa huo? | Ndio: 1  Hapana: 2 | CH2 |
| 41 | ***Kama umejibu NDIO katika swali la 40,*** Je, umeshapatwa na magonjwa zaidi ya moja ya hali hio ndani ya miaka mitatu iliopita? | Ndio: 1  Hapana: 2 | CH3 |
|  | **HISTORIA YA MAGONJWA** |  |  |
| 42 | Ulishawahi kuuguwa/kupatwa, au kujulishwa hospitali kuwa una: |  |  |
|  | 1. Kuumia, au upasuaji unaohusu kifua chako? | Ndio: 1  Hapana: 2 | PA1a |
|  | b) Ugonjwa/matatizo ya moyo? | Ndio: 1  Hapana: 2 | PA1b |
|  | c) Bronchitis? | Ndio: 1  Hapana: 2 | PA1c |
|  | d) Homa ya mapafu (Pneumonia)? | Ndio: 1  Hapana: 2 | PA1d |
|  | e) Pleurisy? | Yes: 1  No: 2 | PA1e |
|  | f) Pumu? | Ndio: 1  Hapana: 2 | PA1f |
|  | g) Magonjwa/matatizo mengine ya kifua? | Ndio: 1  Hapana: 2 | PA1g |
|  | h) Hay fever | Ndio: 1  Hapana: 2 | PA1h |
|  | i) Kifua kikuu (TB) | Ndio: 1  Hapana: 2 | PA1i |
|  | j) Ulanga wa kifua (Silicosis) | Ndio: 1  Hapana: 2 | PA1j |
|  | **UVUTAJI SIGARA** |  |  |
| 43 | Je, unavuta sigara? | Ndio: 1  Hapana: 2 | TS1 |
| 44 | ***Kama umejibu NDIO katika swali la 43,*** Je, umeshawahi kuvuta sigara kwa kiwango cha kuanzia sigara moja kwa siku, kwa muda usiopungua mwaka mmoja? | Ndio: 1  Hapana: 2 | TS2 |
|  | ***Kama umejibu HAPANA kwa swali la 43 au 44, usijibu maswali yanayofuata*** |  |  |
| 45 | Ulikuwa na umri gani ulipoanza kuvuta sigara mfululizo? | ______(*miaka*) | TS3 |
| 46 | Unavuta (Ulikuwa unavuta) sigara za viwandani? | Ndio: 1  Hapana: 2 | TS4 |
| 47 | ***Kama umejibu NDIO kwa swali la 46:*** |  |  |
|  | 1. Je, unavuta (ulikuwa unavuta) sigara ngapi kwa siku? | ______ (*namba*) | TS4a |
|  | b) Siku za wiki (Jumatatu – Ijumaa) | ______ (*namba*) | TS4b |
|  | c) Siku za mwishi wa wiki (Jumamosi – Jumapili) | ______ (*namba*) | TS4c |
| 48 | Je, unavuta aina nyingine yoyote ya tumbaku? | Ndio: 1  Hapana: 2 | TS5 |
| 49 | ***Kama NDIO kwa swali la 48: Toa maelezo katika nafasi ya ‘maelezo ya ziada’*** | *Maelezo ya ziada*:  ____________________________  __________________________  __________________________  __________________________  __________________________  __________________________ | TS6 |
| 50 | Je, lini uliacha kabisa kuvuta? | ______________(*mwezi/mwaka*) | TS7 |

***Additional Notes*** *This questionnaire is based on the MRC (UK) Respiratory Questionnaire 1986, which has been extensively validated. This questionnaire will be completed by a worker with the assistance of a native speaker. Additional questions have been added to cover clinical aspects of bronchial hyper responsiveness validated by the Department of Occupational and Environmental Medicine, National Lung Institute. The British Occupational Health Research Foundation (BOHRF) concluded that in the clinical setting questionnaires that identify symptoms of wheeze and/or shortness of breath which improve on days away from work or on holidays have a high sensitivity, but relatively low specificity for occupational asthma.*

### In-depth Interview Guide – English Version

**IN-DEPTH INTERVIEW GUIDE**

**KEY INFORMANTS - SMALL SCALE TANZANITE MINERS IN MERERANI**

*Copies of informed consent forms should be provided to each participant and read aloud for the benefit of those who cannot read. Participants should be provided an opportunity to ask any questions. Verbal agreement should be taped.*

To start up the discussion, you may want to start the discussion by asking the participant about some of the activities he/she carries out as a SSM, ask about when and how he came to work as a SSM in Mererani and his/her duration of work.

Then explain the ground rules as follows; ‘***Before we proceed further, I would like to make a note that, now I am going to ask you some specific questions related to your work and your lung health, and there are no right or wrong answers in this discussion. We are interested in knowing your personal perceptions on specific question as related to your mining activities and your lung health. So please be relaxed and feel free to be frank and to share your perceptions as this is very important to us so as to learn from you***’

**Demography**

| **Ser No** | **Characteristics** | **Response** |
| --- | --- | --- |
| 1 | Age | __________(Years) |
| 2 | Sex (*by observation*) | __________ (Male/Female) |
| 3 | Religion | __________ |
| 4 | Residency by birth | _______________ (Region)  _______________ (District) |
| 5 | Duration of current occupation | __________ (Years) |
| 6 | Marital status | ____________ (married, widowed, divorced, cohabiting) |

**Guide Questions:**

1. How do you describe the general working environment of the mining pit(s) you have worked for here in Mererani?

**(Probe: How conducive is the working environment to you?, How do you describe your safety in the pits?, Are there sick SSM in your working area?, if so, what forms of sickness are they suffering from?)**

1. What are your perceptions on handling sick workers is organized at your working area?

**(Probe: Where do you get emergency and routine health services? How satisfied are you with provided health services?, How do you cater for the costs for health care services?)**

1. What are the main risk factors to your lung health that are related to your work?

**(Probe: Level of ventilation/air supply in the pits?, Level of exposure to dust within the pits?, Poisonous gases from the underground rocks?, Presence of coughing workers in the same pit?, Presence of known/presumed TB patients in the same working pit?)**

1. (*If has mentioned (or commented after probing) about dust, poor ventilation or poisonous gases as risk factors, then specifically ask*), How do these exposures affects your lungs’ health?

**(Probe: Aware of silica dust? Aware of radon gas?, Meaning of poor/low ventilation/air supply in the working environment down the pit?, How does the lung health affected by one or more of these conditions?, Do you perceive there is one or more of these mentioned risks in your working environment down the pit?)**

1. How do you describe the amount of dust you have inhaled for the time you have been working as a SSM in Mererani?

**(Probe: Use of personal protective equipment against dust inhalation, use of water based drilling for dust reduction, waiting time before entering the pit after blasting)**

1. How do you perceive the quality of ventilation (air supply) from compressors to your working area down the pit?

**(Probe: Amount of air supplied in relation to number of workers?, Cleanliness of the supplied air?)**

1. If ever, what are the signs/symptoms of lungs ill-health you have experienced of which you perceive to be the result of the mining activities?

**(Probe: Shortness of breath, cough, chest pain, and bloody stained sputum)**

1. Have you ever visited a health facility as a result of any of these signs/symptoms? If so, what disease(s) were you told to be suffering from?
2. How do you describe the health services you received and how satisfied with the services were you?

**(Probe: Spirometry for lung function, Sputum test for tuberculosis, chest X-ray)**

1. May you share (if any) experience of your fellow worker suffering from lung disease of which you perceive to be the result of mining activities in Mererani?
2. Let’s summarize some of the key points from our discussion (……...). Is there anything else you would like to add or advise?
3. Do you have any questions or area that you may need me to clarify?

**Thank you for participating in this discussion.**

### In-depth Interview Guide – Swahili Version

**MWONGOZO WA MAHOJIANO YA KINA**

**WAHOJIWA WAKUU – WACHIMBAJI WADOGO WA MADINI YA TANZANITE MERERANI**

*Nakala ya fomu ya kuridhia kushiriki katika utafiti itolewe kwa kila mshiriki na kwa wale wasiojua wasomewe kwa sauti ili waelewe kilichoandikwa. Washiriki wapatiwe nafasi ya kuuliza maswali. Ridhaa ya sauti irekodiwe.*

Kuanzisha mahojiano, unaweza kumuuliza mshiriki kuhusu kazi zake za kila siku kama mchimba madini mdogo, pia waeza muuliza ni lini na kwa njia gani alifikia kufanya kazi ya mchimbaji mdogo Mererani, na muda aliofanya kazi hio.

Muelezee taratibu za mahojiano kama ifuatavyo; ‘***Kabla ya kuendelea na mahojiano, ningependa utambue kuwa nitakuuliza maswali maalumu kuhusiana na kazi yako na afya yako ya mapafu, na hakuna jibu ambalo ni sahihi au jibu la makosa katika mahijiano haya. Tunachohitaji ni maoni yako binafsi katika swali husika kuhusiana na kazi zako za uchimbaji na afya yako ya mapafu. Hivyo tafadhili usiwe na shaka na kuwa huru kutupatia maoni yako ambayo ni muhimu sana kwetu kwakuwa tunataka kujifunza kutoka kwako***’.

**Demografia ya Mshirki**

| **Namba** | **Sifa** | **Jibu** |
| --- | --- | --- |
| 1 | Umri | __________(Miaka) |
| 2 | Jinsia (*kwa kuangalia*) | __________ (Me/Ke) |
| 3 | Dini/ Dhehebu | __________ |
| 4 | Makazi ya kuzaliwa | _______________ (Mkoa)  _______________ (Wilaya) |
| 5 | Muda uliojihusisha na kazi ya sasa | __________ (Miaka) |
| 6 | Hali ya ndoa/mahusiano | ____________ (nimeoa/nimeolewa, mjane/mgane, nimeachika, naishi kinyumba bila ndoa) |

**Maswali ya Mwongozo:**

1. Unaweza ukayaelezea vipi kwa ujumla, mazingira ya kazi katika shimo/mashimo ya uchimbaji Mererani, ambayo umewahi kufanya kazi huko?

**(Vidadisi: Ni kwa namna gani unaridhika na mazingira yako ya kazi?, Unaelezea vipi swala la usalama katika eneo lako la kazi? Kuna wachimbaji wenye matatizo ya kiafya katika mashimo? Kama wapo, ni matatizo gani ya kiafya walionayo?, Upatikanaji wa huduma za afya katika eneo la kazi?)**

1. Nini maoni yako kuhusu jinsi utaratibu wa kuwahudumia wafanyakazi wagonjwa unavyoratibiwa katika eneo lako la kazi?

**(Vidadisi: Wapi unapata huduma za afya za dharura na za muda mrefu?, Ni kwa kiasi gani unaridhishwa na huduma za afya zitolewazo?, Ni kwa vipi unalipia gharama za matibabu yako?)**

1. Ni vitu/mazingira gani katika mazingira yako ya kazi ambavyo ni hatarishi zaidi kwa afya yako ya mapafu?

**(Vidadisi: Kiwango cha mzunguko wa hewa kwenye mashimo?, Kiwango cha vumbi katika mashimo? Gesi za sumu kutoka kwenye miamba ya mashimo?, Kuwepo kwa wachimbaji wanaokohoa kwenye mashimo?, Kuwepo kwa wachimbaji wanaojulikana au kudhaniwa kuwa na kifua kikuu?)**

1. *(Kama ametaja (au kuafiki baada ya udadisi) kuhusu vumbi, mzunguko finyu wa hewa katika mashimo au gesi za sumu, uliza)*; Elezea ni kwa jinsi gani uwepo wa vumbi, mzunguko finyu wa hewa katika mashimo au gesi za sumu kunaathiri afya ya mapafu yako?

**(Vidadisi: Waelewa nini kuhusu vumbi ya silica?, Waelewa nini kuhusu gesi ya radon? Nini maana ya kuwa na mzunguko finyu wa hewa katika mazingira ya kazi kwenye mashimo?, Afya ya mapafu inaathirikaje kwa uwepo wa hali moja au zaidi ya hizi tajwa? Unadhani katika mazingira yako ya kazi kuna hali yeyote kati ya hizi tajwa?-Elezea)**

1. Nini maoni yako kuhusu kiwango cha vumbi ambacho unaweza kuwa umevuta kupitia mfumo wako wa hewa kwa muda wote uliofanya kazi ya uchimbaji?

**(Vidadisi: Matumizi ya vifaa vya kujikinga na kuvuta vumbi?, Matumizi ya maji ili kupunguza vumbi wakati wa uchimbaji?, Muda wa kusubiri kabla ya kuingia shimoni baada ya kupasua miamba kwa baruti?)**

1. Una maoni gani kuhusu ubora wa hewa inayosukumwa katika shimo?

**(Vidadisi: Kiwango/ ujazo wa hewa kwa kilinganisha na idadi ya wachimbaji walio katika shimo?, Usafi wa hewa inauyosikumwa chini katika shimo?)**

1. Kama ilishakutokea, ni zipi dalili ya kuarithika kwa mapafu ambazo ushawahi kuzipata na unadhani zinatokana na kazi yako ya uchimbaji?

**(Vidadisi: Kubanwa na pumzi?, Kukohoa?, Maumivu ya kifua?, Kukohoa makohozi yenye damu?)**

1. Ulishawahi kwenda hospitali kutokana na dalili yoyote kati ya ulizotaja? Kama jibu ni ndiyo, je ulijulishwa kuwa una ugonjwa gani?
2. Una maoni gani kuhusu huduma uliopatiwa hospitalini na je uliridhishwa na huduma hizo?

**(Vidadisi: Uchunguzi wa ufanyaji kazi wa mapafu?, Uchunguzi wa kifua kikuu kupitia makohozi?, Uchunguzi wa kifua kupitia X-ray?)**

1. Tupatie uzoefu wako (kama upo) kuhusu mchimbaji mwenzako ambae ameshapatwa na athari/ugonjwa wa kifua/mapafu ambao unadhani umetokana na kazi ya uchimbaji Mererani.
2. Kwa sasa tuhitimishe kwa kupitia vitu muhimu katika mahojiano yetu (………). Kuna lolote ungependa kuongeza au kushauri?
3. Je, una swali lolote au kipengele cha mahojiano yetu ambacho ungependa nitolee maelezo?

**Asante kwa kushiriki katika mahojiano haya.**

## Consent Forms

### General Consent Form – English Version

SILICA AND RADON EXPOSURES AND ITS ASSOCIATED EFFECTS ON RESPIRATORY SYSTEM AMONG SMALL SCALE TANZANITE MINERS IN MERERANI

Study Participant Consent Form

**I, _______________________________________________,** I do consent by my own will, to participate in the research project titled;

**‘Silica and Radon Exposures and Its Associated Effects on Respiratory System Among Small Scale Tanzanite Miners in Mererani’**

**I acknowledge that:**

I have been well informed about the study and given enough time to ask questions and I have been satisfied with provided responses. This includes that I will be asked some questions as per the prepared ‘interview schedule’, I may be requested to be fitted with a device (personal dust sampler) during on of the days of the study for collecting samples of air around my working area that will later be used to determine the presence of respirable crystalline silica and I will have to wear this device throughout my working shift on that day. Also, as per the study schedule, I may be asked to have one day when I will have to travel to Kibong’oto Infectious Diseases Hospital (KIDH) in Siha District, using a transport prepared by the investigator, to undergo spirometry test for lung function assessment and also to be screened and investigated of tuberculosis (TB) through sputum investigation and chest X-ray. After completion of the investigations, I will be travelling back to Mererani using a prepared transport by the investigator.

I consent to participate:

☐ By wearing an air sampling pump to measure the silica I am breathing

☐ By responding to the questions as per the interview schedule

☐ By allowing myself to be filmed while doing my work for body positioning improvements.

I understand that my involvement is voluntary and that the information gained during the study may be published but no information about me will be used in any way that reveals my identity. I understand that I can withdraw from the study at any time without explanation.

Signature__________________________________________

Names (Three)_____________________________________

Date______________________________________________

**Contacts:**

1. **Investigator: Alexander William Mbuya, P O Box 12, Sanya Juu, Mobile number: +255 754812626**
2. **College Research and Ethical Review Committee of Kilimanjaro Christian Medical University College: P O Box 2240, Moshi. Simu namba: +255272754377/83**

### General Consent Form – Swahili Version

**FOMU YA RIDHAA YA KUSHIRIKI KATIKA UTAFITI**

**Uvutaji wa Vumbi la Silica na Gesi ya Radon na Madhara Yake Katika Mfumo wa Upumuaji kwa Wachimbaji Wadogo wa Madini ya Tanzanite, Mererani.**

**Mimi,__________________________________________________,** ninakubali kwa maamuzi yangu binafsi na bila kushurutishwa, kushiriki katika utafiti huu unaohusiana na, **Uvutaji wa Vumbi la Silica na Gesi ya Radon na Madhara Yake Katika Mfumo wa Upumuaji kwa Wachimbaji Wadogo wa Madini ya Tanzanite, Mererani.**

**Ninakubali kwamba:**

Nimeelimishwa kuhusu utafiti huu na kupewa nafasi ya kuuliza maswali kuhusu ushiriki wangu na kupatiwa majibu yaliyoniridhisha. Hii ni pamoja na kuwa nitaulizwa maswali kuendana na dodoso (interview schedule) lililoandaliwa, naweza kuombwa kuvalishwa kifaa maalum (personal dust sampler) katika moja ya siku wakati wa utafiti huu ambapo nitakivaa kwa wakati wote wa muda wangu wa kazi zangu za kawaida ili kunasa sampuli za hewa zitakazotumika kuchunguza kiasi cha vumbi la silica lililopo katika mazingira yangu ya kazi. Pia kuendana na ratiba ya utafiti huu, nitapata siku moja ambayo usafiri utaandaliwa na mtafiti ili kuniwezesha kufika katika Hospitali ya Magonjwa Ambukizi ya Kibong’oto (Wilaya ya Siha), ili kufanyiwa uchunguzi wa ufanyaji kazi wa mfumo wa mapafu na pia kuchunguzwa uwepo wa ugonjwa wa kifua kikuu kupitia makohozi na X-ray ya kifua. Baada ya kukamilisha vipimo hivo nitapatiwa majibu yangu na kurejeshwa Mererani kwa usafiri ulioandaliwa na mtafiti.

Ninatoa idhini ya ushiriki:

☐ Kwa kuvaa pampu ya kuchukulia sampuli ya hewa kupima kiwango cha vumbi la silica ninalovuta

☐ Kwa kujibu maswali nitayoulizwa kuendana na dodoso.

☐ Kuruhusu kuchukua/kurekodi picha na video kipindi nafanya kazi zangu kuangalia jinsi ya kuboresha namna sahihi ya kukaa na kufanya kazi katika mikao salama.

Ninatambua kuwa ushiriki wangu ni wa hiari na taarifa zote zitakazopatikana wakati wa utafiti huu zinaweza kutumika katika kuelimisha jamii kuhusu tafiti hii, ikiwemo kuchapishwa katika majarida mbalimbali bila kuweka taarifa za afya yangu binafsi au utambulisho katika majarida hayo. Ninatambua ya kuwa nina uwezo wa kujitoa katika tafiti hii muda wowote, bila kutoa maelezo yoyote ya awali toka kwangu.

Sahihi______________________________________________

Jina (Matatu)________________________________________

Tarehe______________________________________________

**Mawasiliano:**

1. **Mtafiti mkuu: Alexander William Mbuya, S. L. P. 12, Sanya Juu, Simu namba: +255754812626**
2. **Kamati ya Mapitio ya Tafiti na Madili ya Chuo Kikuu cha Kikristu cha Tiba Kilimanjaro, S. L. P 2240 Moshi. Simu namba: +255272754377/83**

### In-depth Interview Consent Form – English Version

**CONSENT FORM FOR STUDY PARTICIPATON**

**Silica and Radon Exposures and Its Associated Effects on the Respiratory System Among Small Scale Miners in Mererani**

**In-Depth Interview**

**KILIMANJARO CHRISTIAN MEDICAL UNIVERSITY COLLEGE**

**GOAL**: The goal of this study is explore your awareness and perceptions about exposure to silica dust and radon gas and its associated effects in the respiratory system (lungs). I am conducting this study so as to understand those effects and hence being able to recommend on preventive measures and also I am using this study as part of my training program for the degree of Doctor of Philosophy.

**PARTICIPATION:** If you consent to participate in this study, you will answer/respond to already prepared questions. Your participation is voluntary and if you decide not to participate, then your decision will not affect by any means the kind and quality of services you are supposed to get.

**CONFIDENTIALITY:** The information that you will provide us will remain confidential and will be used for the purpose of this study only. In addition, in this study you will not be identified by your actual name but by a code.

**RISKS:** It may occur that some questions will remind you of some unpleasant memories, but we don’t expect you to get any significant harm from your participation.

**RIGHT TO WITHDRAW**: If you decide to participate, and for any reason during the, you decided that you don’t want to proceed with the study; you can decide to withdraw at any time and without providing any reason for your decision.

**BENEFIT:** There is no direct benefit from participation but the obtained information from this study will be used to design programs that will help in improving the health of mine workers in general.

**CONTACTS**: In case you may need more clarification or information about this study, don’t hesitate to contact one of:

1. **Investigator: Alexander William Mbuya, Kilimanjaro Christian Medical University College, P O Box 2240 Moshi, Mobile: +255754812626**
2. **College Research and Ethical Review Committee of Kilimanjaro Christian Medical University College: P O Box 2240, Moshi. Phone no: +255272754377/83**

**AGREEMENT**

I, …………………………………………….have read the what has been written in this consent form and understood. Any question(s) I had have been responded to and I am satisfied.

I do consent to participate in this study.

Participant signature:

…………………...................................

Investigator signature/ Assistant investigator signature:

……………..........................................

Date:

…………..............................................

### In-depth Interview Consent Form – Swahili Version

**FOMU YA RIDHAA YA KUSHIRIKI KATIKA UTAFITI**

**Uvutaji wa Vumbi la Silica na Gesi ya Radon na Madhara Yake Katika Mfumo wa Upumuaji kwa Wachimbaji Wadogo wa Madini ya Tanzanite, Mererani.**

**Mahojiano ya Kina**

**CHUO KIKUU CHA KIKRISTU CHA TIBA KILIMANJARO**

**DHUMUNI**: Utafiti huu una lengo la kubaini uelewa na maoni yako kuhusu uvutaji wa vumbi yenye madini ya silica (ulanga) na gesi ya ‘radon’ na athari zake katika mfumo wa hewa wa binadamu (mapafu). Ninafanya utafiti huu ili kuweza kuelewa athari hizo na kutoa ushauri jinsi ya kuzuia athari hizo na pia natumia utafiti huu kama sehemu ya programu ya mafunzo yangu ya shahada ya uzamivu.

**USHIRIKI:** Endapo utakubali kushiriki katika utafiti huu, utajibu maswali yalioandaliwa. Ushiriki wako ni wa hiari na endapo utaamua kutokushiriki, uamuzi huo hauta punguza au kuathiri kwa vyovyote vile matibabu au huduma zingine utakazohitaji kupatiwa.

**USIRI:** Taarifa zote utakazozitoa zitabaki kuwa siri na zitatumika kwa ajili ya utafiti huu pekee. Pia katika utafiti huu hautatambulika kwa jina lako halisi bali itatumika namba maalumu kutambua maoni yako.

**MADHARA:** Yawezekana baadhi ya maswali yakakuletea kumbukumbu usioipenda kama vile hivo kugusa hisia zako, lakini hatutegemei upate madhara yoyote makubwa kwa kushiriki kwako.

**HAKI YA KUJITOA**: Ushiriki katika utafiti huu ni hiari, hivo una hiari ya kushiriki ama kutoshiriki. Ukiamua kushiriki, na kwa sababu yeyote ile ukaona hupendi kuendelea kushiriki, unaweza kutoendelea muda wowote na bila kutoa maelezo yoyote.

**FAIDA:** Hakutakuwa na faida ya moja kwa moja ila taarifa utakayo tupatia katika utafiti huu, itatumika katika kupanga mipango itakayosaidia kuboresha afya za wachimba madini kwa ujumla.

**MAWASILIANO**: Endapo utahitaji maelezo zaidi au taarifa kuhusiana na utafiti huu, usisite kuwasiliana na mmojawapo kati ya:

1. **Mtafiti mkuu, Dk. Alexander William Mbuya wa Chuo Kikuu cha Kikristu cha Tiba Kilimanjaro, S. L. P. 12 Sanya Juu, Simu namba: +255754812626**
2. **Kamati ya Mapitio ya Tafiti na Maadili ya Chuo Kikuu cha Kikristu cha Tiba Kilimanjaro, S. L. P 2240, Moshi. Simu namba: +255272754377/83**

**MAKUBALIANO**

Mimi …………………………………………………nimesoma yaliyo andikwa katika fomu hii ya ridhaa na kuyaelewa. Maswali yangu yamejibiwa na nimeridhika. Ninakubali kwa hiari yangu kushiriki katika utafiti huu.

Sahihi ya Mshiriki:

…………………...................................

Sahihi ya MtafitiMkuu/ Msaidizi wa Mtafiti Mkuu:

……………..........................................

Tarehe:

…………..............................................
